# Studies of Nature of Uncommon Bifurcated I–I···(I–M) Metal-Involving Noncovalent Interaction in Palladium(II) and Platinum(II) Isocyanide Cocrystals

**DOI:** 10.1021/acs.inorgchem.1c01591

**Published:** 2021-08-06

**Authors:** Margarita Bulatova, Daniil M. Ivanov, J. Mikko Rautiainen, Mikhail A. Kinzhalov, Khai-Nghi Truong, Manu Lahtinen, Matti Haukka

**Affiliations:** † Department of Chemistry, University of Jyväskylä, P.O. Box 35, FI-40014 Jyväskylä, Finland; ‡ Institute of Chemistry, Saint Petersburg State University, Universitetskaya Nab. 7/9, Saint Petersburg 199034, Russian Federation

## Abstract

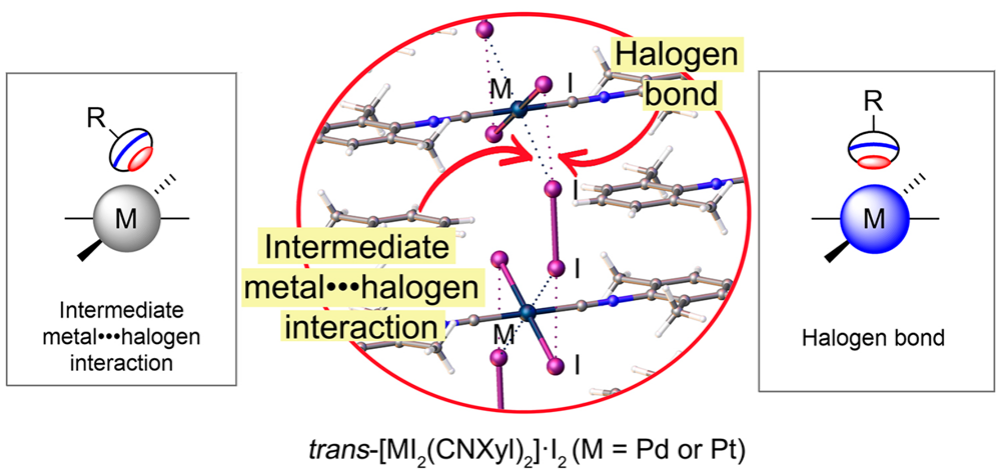

Two isostructural *trans*-[MI_2_(CNXyl)_2_]·I_2_ (M = Pd or Pt; CNXyl = 2,6-dimethylphenyl isocyanide) metallopolymeric cocrystals containing uncommon bifurcated iodine···(metal–iodide) contact were obtained. In addition to classical halogen bonding, single-crystal X-ray diffraction analysis revealed a rare type of metal-involved stabilizing contact in both cocrystals. The nature of the noncovalent contact was studied computationally (via DFT, electrostatic surface potential, electron localization function, quantum theory of atoms in molecules, and noncovalent interactions plot methods). Studies confirmed that the I···I halogen bond is the strongest noncovalent interaction in the systems, followed by weaker I···M interaction. The electrophilic and nucleophilic nature of atoms participating in I···M interaction was studied with ED/ESP minima analysis. In *trans*-[PtI_2_(CNXyl)_2_]·I_2_ cocrystal, Pt atoms act as weak nucleophiles in I···Pt interaction. In the case of *trans*-[PdI_2_(CNXyl)_2_]·I_2_ cocrystal, electrophilic/nucleophilic roles of Pd and I are not clear, and thus the *quasimetallophilic* nature of the I···Pd interaction was suggested.

## Abstract 2

Metal···halogen interaction of intermediate nature between halogen and semicoordination bond was discovered for isostructural cocrystals *trans*-[MI_2_(CNXyl)_2_]·I_2_ (M = Pd or Pt; CNXyl = 2,6-dimethylphenyl isocyanide). The geometrical parameters of the metal···halogen interaction obtained from SCXRD analysis raised the question of the polarity of the interaction. Further computational investigations revealed the difference between the I···Pd contact, which was found to be nonpolar or *quasimetallophilic*, and the I···Pt contact, which is weakly polar, metal-involving halogen bonding.

## 1 Introduction

**Figure 1:**
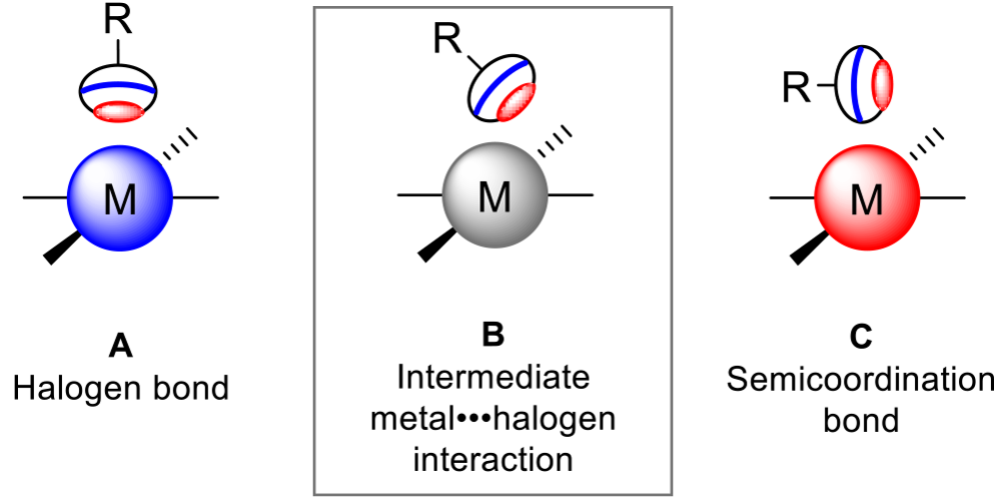
Types of NCIs involving metal centers (M) and halogen atoms (drawn as black ovals), where electrophilic regions are colored as red and nucleophilic ones are colored as blue: metal-involving halogen bond (A); intermediate metal–halogen interaction (B); semicoordination bond (C).

Noncovalent interactions (NCIs) are a powerful instrument applied in such fields as synthesis,^1^ catalysis,^2,3^ design of photoactive materials,^4−6^ and biochemistry.^7,8^ Halogen bonding (XB), in particular, has been found to be a very useful NCI, for example, in the synthesis of self-assembled polymers,^9,10^ due to its high directionality and possibilities for fine-tuning. Recently, XB has been utilized in our research to create metallopolymeric systems.^11^ Known types of metal–halide interactions involved in the self-assembly of metallopolymers include classical XB^12^ (Figure 1A) and semicoordination bond via electron belt (Figure 1C).

In cocrystals of metal complexes, classical XB is represented by donor/acceptor interaction of an electron-deficient area (σ-hole) located on a XB donor (XBD) and an electron-rich area located either on a ligand or on the metal center itself. In the case of an interaction with square planar d^8^, linear d^10^ transition metal complexes, or metal surface, an electron lone pair on the d orbital acts as the nucleophile, while a σ-hole of a halogen atom acts as the electrophile. The first examples of the possible metal-involving XBs were represented by van Koten et al.^13−16^ for the I–I···Pt^II^ bonds between diiodine and NCN pincer Pt^II^ complexes. Theoretical investigations of these interactions showed that they are rather strong and comparable with coordinative bonds.^17,18^ Further works of van Koten et al. showed that the analogous palladium and nickel NCN pincer complexes interact with diiodine in other ways.^19−21^ Nevertheless, later works represented metal-involving XB not only with Pt^II^^22−28^ and Pd^II^^23,27,29,30^ but also with Ni^II^,^27,31^ Rh^I^,^32,33^ Au^I^,^34−36^ and Au^0^ centers^37−41^ as nucleophiles.

In contrast to XB, a semicoordination bond^31^ occurs when an electrophilic region of a metal center is interacting with the electron belt of a halogen atom or a nucleophilic halide anion. Particularly, examples of Pd^II^···I^31,42−47^ and Pt^II^···I^48−50^ semicoordination bonds have been described in the literature.

Both types of discussed noncovalent interactions between metal centers and halogen atoms can be considered *polar* NCIs (with clear electrophilic or nucleophilic^51^ roles assignable to interacting atoms). In this connection, it is worth noting the well-defined *nonpolar* NCIs (with unclear electro- or nucleophilic roles) between the halogen atoms (type-I halogen···halogen interactions caused by dispersive forces)^3^ and metallophilic interactions (closed-shell (d^10^, s^2^) or pseudoclosed shell (d^8^) weak attractive metal···metal contacts presumably dominated by electrostatic and dispersion forces).^52^ Although possible a *nonpolar* NCI involving metal center and halogen atoms is mentioned for the so-called C–I···Ni boundary case,^31^ the nature of *nonpolar* interaction (such as philicity of interacting centers and energy components) between halogen and metal atoms has never been studied thoroughly prior to this work.

As a continuation of our studies of metal-involving interactions^53^ and halogen bonding,^26^ especially between molecular iodine and iodide isocyanide complexes,^53,54^ the association of molecular iodine with *trans*-[MI_2_(CNXyl))_2_] (M = Pd (**1**) or Pt (**2**); CNXyl = 2,6-dimethylphenyl isocyanide) species was studied. The simple structure of molecular iodine allows a high level of control in the self-assembly of noncovalently bound metallopolymers. Bearing both an electron-deficient region of a σ-hole and an electron-rich area of an electron belt, I_2_ is prone to interact with both electrophilic and nucleophilic regions of other molecules.^55^ In addition, the relatively small size of the I_2_ molecule allows it to overcome steric constraints. Furthermore, square planar *trans*-[MI_2_(CNXyl)_2_] are promising building blocks in organometallic chemistry due to stabilizing metal–carbon π interactions.^56,57^ An occupied d_*z*^2^_ orbital of these Pd and Pt complexes is accessible for interaction, which opens up the opportunity for the generation of metal–involving XB systems.

**Figure 2:**
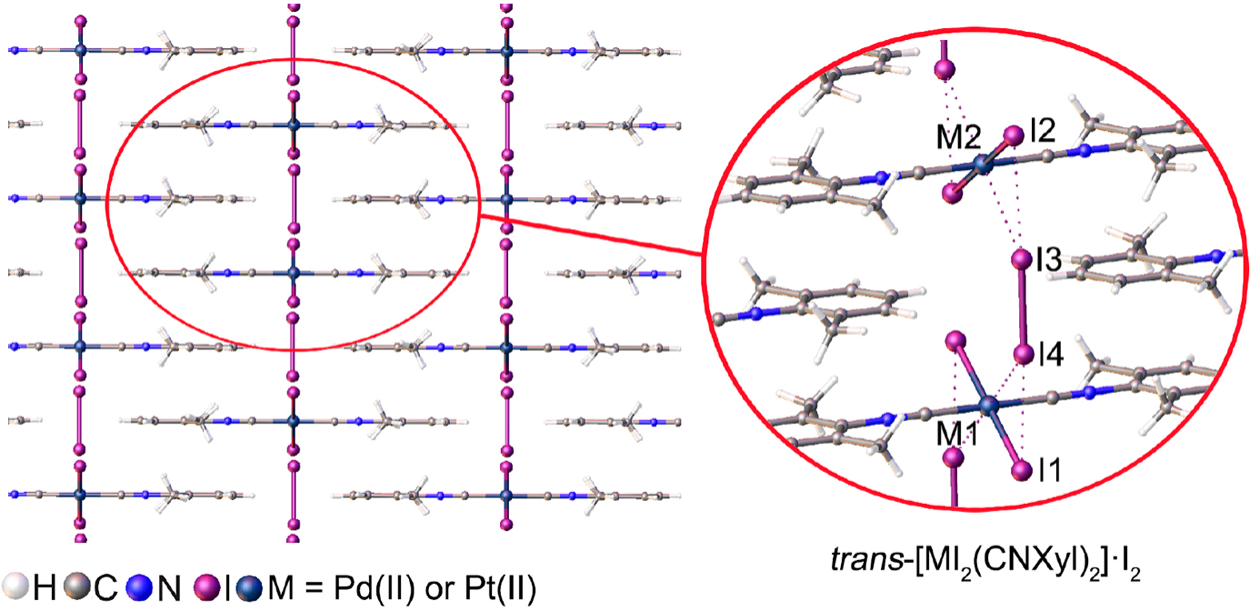
Representations of *trans*-[MI_2_(CNXyl)_2_]·I_2_ (M = Pd or Pt) polymeric crystal structures visualizing π–π stacking (left) and noncovalent bifurcated I–I···(I–M) contact (right) in both cocrystals along the *a-*axis.

In the current work, molecular iodine forms isostructural metallopolymeric cocrystals, *trans*-[MI_2_(CNXyl)_2_]·I_2_ (M = Pd or Pt), where complex units are noncovalently linked via I_2_ molecules (Figure 2). Careful analysis of experimental and theoretical data along with a literature search revealed atypical I–I···(I–M) bifurcated noncovalent bonds, in which classical halogen bond is additionally stabilized by an uncommon type of an I···M contact between a metal center and halogen atom. In this contact, the halogen atom is neither interacting via a σ-hole (Figure 1A) nor via an electron belt (Figure 1C), but presumably via a transitional area (Figure 1B). To understand the nature of this intermediate contact, it was comprehensively studied with various bond analysis methods such as electrostatic surface potential (ESP) analysis (to discover the angle limits of a σ-hole), NCIs plot (NCI-plot) analysis (to reveal the relative strength of the interaction), electron density (ED)/ESP analysis (to assign philicity of the interacting atoms), and local energy decomposition (LED) analysis (to indicate which interaction type best describes the contact).

## 2 Results and Discussion

### 2.1 Complexes **1** and **2** and Their Cocrystals

**Scheme 1:**
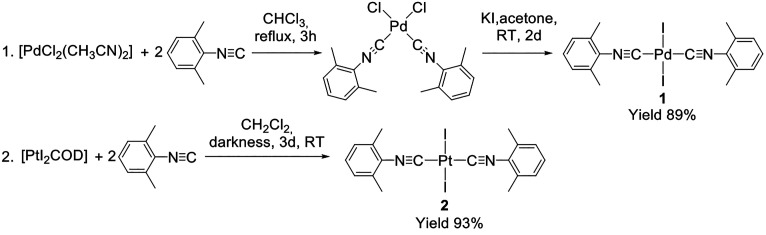
Synthesis of Complexes **1** and **2**

Syntheses of complexes *trans*-[PdI_2_(CNXyl)_2_]^58^ (**1**) and *trans*-[PtI_2_(CNXyl)_2_]^59^ (**2**) are presented in Scheme 1. A similar *trans*-[PdBr_2_(CNXyl)_2_] complex^60^ has been described previously. Cocrystals **1**·I_2_ and **2**·I_2_ were grown from 1:1 CH_2_Cl_2_/CHCl_3_ and CHCl_3_ solutions of a 1:1 mixture of the corresponding complex and I_2_, respectively.

### 2.2 Single-Crystal X-ray Diffraction (SCXRD) Analysis

Although syntheses of **1**(58) and **2**(61) are known, no SCXRD data was found for these complexes in the Cambridge Structural Database (CSD). Isostructural complexes **1** and **2** exhibit the same monoclinic lattice of the *P*2_1_/*c* space group (for details, see the Supporting Information, “Single crystal X-ray Diffraction Data Analysis (SCXRD)” section). The complexes have square-planar structures with iodide ligands in the *trans* position to each other. Cocrystals of **1**·I_2_ and **2**·I_2_ have both a 1:1 molar composition; exhibit the same triclinic lattice *P*1̅ space group, and similar unit cell parameters, being isostructural to the original complexes. The fragments C–N–C–M are almost linear in both complexes (∠(Pd–C–N) = 179.2(4)° and ∠(Pt–C–N) = 178.6(4)°) and corresponding cocrystals (∠(Pd–C–N) = 179.4(10)° and ∠(Pt–C–N) = 179.0(12)°). The most notable difference between original **1** and **2** complexes and the corresponding cocrystals is in the position of the iodide ligands: While in **1** and **2** the iodide ligands are located in the same plane as the xylene rings of the CNXyl ligands, in cocrystals the iodide ligands are tilted away from the plane allowing interaction of I_2_ molecule with the complex. Additionally, in cocrystals CNXyl ligands are arranged in π–π stacks with centroid–centroid distances of 3.86–3.88 Å in **1**·I_2_ and 3.87–3.91 Å in **2**·I_2_ (Figure 2), whereas in original **1** and **2** complexes, this type of stacking is not observed.

The relative strengths of NCIs can be approximated by a comparison of the experimentally obtained distances between noncovalently interacting atoms and the sum of corresponding van der Waals radii (vdW).^62^ The distance reduction ratio of NCI (*R*_IX_) can be calculated as *R*_IX_ = *d*(I···X)/(*R*^I^_vdW_ + *R*^X^_vdW_), where I (iodine) represents a halogen bond donor (XBD) atom, X is a halogen bond acceptor (XBA) atom, *d*(I···X) is the distance between I and X in Å, and *R*^I^_vdW_ and *R*^X^_vdW_ are the vdW radii by Bondi^63^ of I and X in Å, respectively. The comparison shows that the characteristic parameters of the interactions correlate closely with each other emphasizing the isostructural nature of cocrystals (Figure 2, Table 1).

**Table 1:** Characteristic Parameters of Selected Noncovalent Interactions in the Crystal Structures of **1**·I_2_ and **2**·I_2_

cocrystal	contact				
	I···I				
	I–I···I–Pd	*d*(I···I), Å	∠(I–I···I), deg	∠(I···I–Pd), deg	*R*_IX_^a^
**1**·I_2_	I3–I4···I1–Pd1	3.4986(11)	173.07(3)	65.82(2)	0.88
	I4–I3···I2–Pd2	3.5034(11)	173.10(3)	65.74(2)	0.88

	I···Pd				
	I–I···Pd–I	*d*(Pd···I), Å	∠(I–I···Pd), deg	∠(I···Pd–I), deg	*R*_IX_^a^
**1**·I_2_	I3–I4···Pd1–I1	3.4038(8)	128.58(3)	65.82(2)	0.94
	I4–I3···Pd2–I2	3.4038(8)	128.64(3)	65.74(2)	0.94

	I···I				
	I–I···I–Pt	*d*(I···I), Å	∠(I–I···I), deg	∠(I···I– Pt), deg	*R*_IX_^a^
**2**·I_2_	I3–I4···I1–Pt1	3.5195(9)	172.13(3)	66.875(17)	0.89
	I4–I3···I2–Pt2	3.5206(9)	172.31(3)	66.751(17)	0.89

	I···Pt				
	I–I···Pt–I	*d*(Pt···I), Å	∠(I–I···Pt), deg	∠(I···Pt–I), deg	*R*_IX_^a^
**2**·I_2_	I3–I4···Pt1–I1	3.4648(6)	128.10(3)	69.092(17)	0.93
	I4–I3···Pt2–I2	3.4601(6)	128.27(3)	69.207(17)	0.93

a *R*_IX_ = *d*(I···X)/(*R*^I^_vdW_ + *R*^X^_vdW_), where *R*_IX_ is distance reduction ratio, I is a donor atom, X is an acceptor atom (I, Pt, Pd), and *d*(I···X) is the distance between I and X in Å; *R*^I^_vdW_ and *R*^X^_vdW_ are the vdW radii of I and X correspondingly determined by Bondi.^63^

The uncommon bifurcated I–I···(I–M) contact can be subdivided into two types of NCIs: I···I and M···I. Within the cocrystals, the relative strength of the XB is rather similar: For I···I XB, *R*_IX_ is 0.88 for **1**·I_2_ and 0.89 for **2**·I_2_; for M···I interaction, *R*_IX_ is 0.94 for **1**·I_2_ and 0.93 for **2**·I_2_. Hence, in both cocrystals I···I XB is slightly stronger than M···I interaction. This might indicate the main role of I···I XB in the interaction (which is further confirmed in theoretical analysis of the structures, *vide infra*). Another parameter attracting attention is the ∠I–I···M angle in both cocrystals, which is significantly more acute (about 128°) than that of classical XB (180°)^12^ (Table 1). Variation of this parameter brings up a question on the nature of I···M interaction; thus, it was further studied computationally.

The I···Pd distances in **1**·I_2_ are shorter by ∼0.06 Å than the same I···Pt distances in **2**·I_2_ (Table 1). At the same time, the electron density values in the corresponding I···M bond critical points (BCPs) are similar (the difference is less than 0.001 a.u., see Table 3 in section 2.3.2). According to these observations, the vdW Pd radius may be similar or only slightly shorter than the Pt radius. This hypothesis is in disagreement with Bondi’s vdW radii (1.63 Å for Pd vs 1.75 Å for Pt),^62^ and further detailed studies should be carried out in this direction.

Comparing M–I bond length in the cocrystals and in the corresponding complexes (Table 2), we observed elongation of the M–I bond in cocrystals, presumably due to a strong influence of the halogen bonding with I_2_ in the cocrystals. This elongation was also found in a few other cocrystals of square planar Pt complexes having different XBDs,^22,64^ and the M–I bond elongation is likely to be found in similar systems of square planar transition metal complexes interacting with XBD.

**Table 2:** M–I and I–I Distances in the Single Crystals of the *trans*-[MI_2_(CNXyl)_2_] Complexes, Corresponding Cocrystals, and I_2_ Molecule^65^

	**1**	**1**·I_2_	**2**	**2**·I_2_	I_2_
*d*(M–I), Å	2.5950(4)	2.6156(7)	2.6028(4)	2.6179(6)	
		2.6158(7)		2.6187(6)	
*d*(I–I), Å		2.7264(9)		2.7400(11)	2.7179(2)

The I–I bond length is elongated in **1**·I_2_ and **2**·I_2_ cocrystals in comparison to that in the solid-state structure of I_2_^65^ (Table 2). This elongation of a covalent bond in XBD is typical for a XB according to IUPAC XB definition.^12^

Although according to *R*_IX_ value halogen bonding seems to be the strongest NCI, it is important to take into consideration the combination of all the involved NCIs like I···M interaction and π–π stacking. The significance of the discussed interactions for the structure arrangement was further elucidated by various computational methods (see the “Theoretical Studies” section and the “QTAIM Analysis” section of the Supporting Information, where QTAIM = quantum theory of atoms in molecules).

### 2.3 Theoretical Studies of Noncovalent Interactions

With the help of computational chemistry, the nature and relative strength of NCIs discovered by SCXRD can be thoroughly studied. Careful analysis of the calculated electron density distribution can reveal NCIs and their properties. A combination of several approaches such as analysis of ESP,^66,67^ NCI-plot^68^ analysis, combined electron localization function (ELF)^69^ and Bader’s quantum theory of atoms in molecules (QTAIM) analysis,^70^ and LED^71^ analysis gives a broad look on NCIs. To support the idea that observed interactions are not only caused by packing effects, the data of single-point (SP) structures and optimized (OPT) ones were compared (for more details, see the Experimental Section).

#### 2.3.1 ESP Analysis

Observed NCIs can be clarified by analysis of anisotropic charge distribution, which is visualized by ESP.^3,67,72−76^ ESP visualizes electron-rich and -deficient areas of the molecule that are likely to participate in electrostatic intermolecular interactions. This helps to estimate the geometries and expected strengths of the XB interactions. The strength of the XB formed by the XBD is related to the magnitude of σ-hole^77^ on the XBD atom that can be described by the maximum of ESP (*V*_s,max_). The influence of the XBA on the XB can be estimated using the minimum of ESP (*V*_s,min_) on the XBA atom electron density surface. ESP analysis was carried out on the 0.001 a.u. contour of molecule’s electron density (that encompasses 96% of the molecular charge).^78^

**Figure 3:**
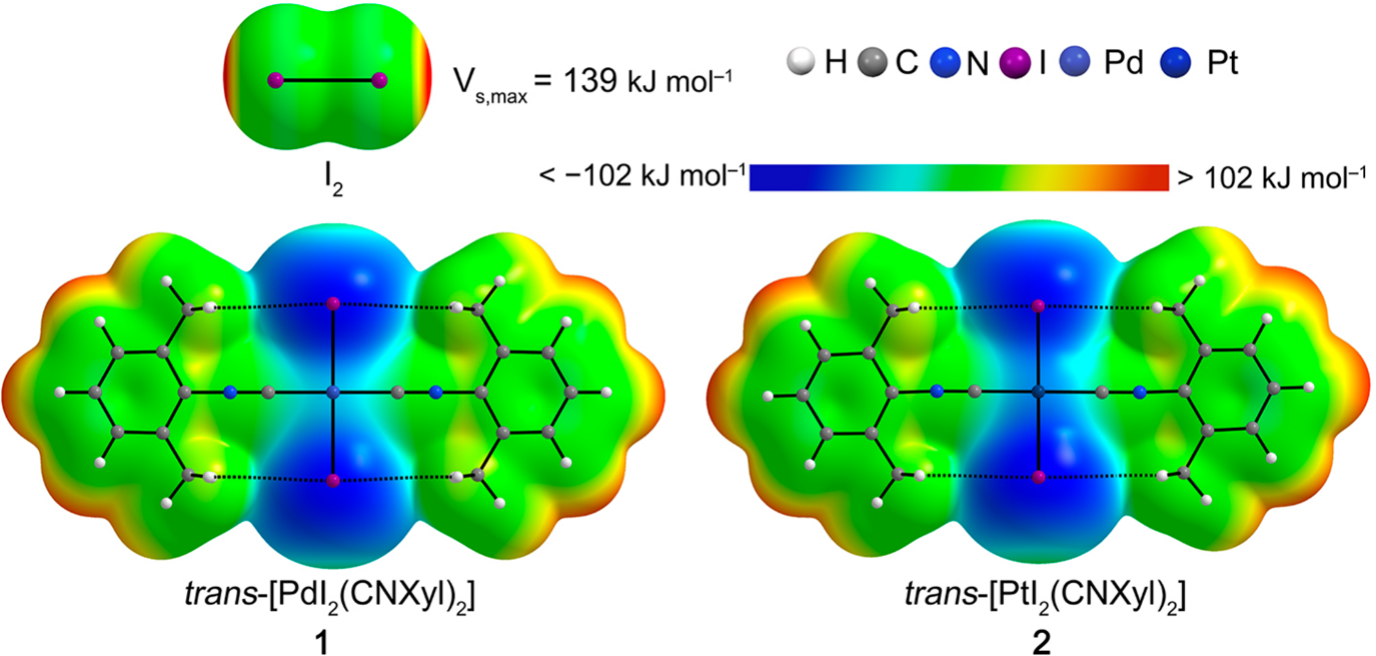
Electrostatic potential calculated at the M06L/def2TZVP/def2TZV computational level on the 0.001 a.u. molecular surface of I_2_, **1**, and **2** with the color scale from −102 kJ mol^–1^ to 102 kJ mol^–1^.

Anisotropic charge distributions of I_2_, *trans*-[PdI_2_(CNXyl)_2_] (**1**), and *trans*-[PtI_2_(CNXyl)_2_] (**2**) were analyzed, and the corresponding ESPs are represented in Figure 3. An electron-deficient area corresponding to the σ-hole of the I_2_ molecule was calculated with *V*_s,max_ = 139 kJ mol^–1^ which is reasonably close to the *V*_s,max_ value (127 kJ mol^–1^) reported by Kolář et al.^79^ at much higher ab initio QCISD/def2-QZVP level of theory. As was suggested by the X-ray diffraction analysis, the I_2_ molecule is expected to behave in the cocrystals as a XBD, interacting with complexes **1** or **2** that act as XBAs. To participate in XB, complexes **1** and **2** are required to bear an electron-rich area around the I or M (M = Pd or Pt) atom. Indeed, ESP studies of **1** and **2** confirm the electron-rich areas (*V*_s,min_) for iodine atoms (*V*_s,min_ = −102 kJ mol^–1^) and for the Pd (*V*_s,min_ = −81 kJ mol^–1^) and Pt centers (*V*_s,min_ = −89 kJ mol^–1^). These local nucleophilic areas roughly correlate with the regions of I···I and I···M interactions.

To analyze if the uncommon I···M contact could be caused by XB-type interactions, we determined the σ-hole limiting angle^80^ [∠(I–I···XBA)] for the I_2_ molecule. The σ-hole limiting angle helps to estimate the angle range where nucleophilic atom can approach the electron-deficient area of I atom with a favorable electrostatic attraction. The limits of the interaction with the σ-hole were found to be 115–180° (see Figure S4). In the case of **1**·I_2_ and **2**·I_2_ cocrystals, ∠(I–I···M) is around 128° allowing M atoms to interact with the σ-holes of I_2_. While this provides evidence of the likely existence of I···M interaction, it does not give direct information on the nature of the interaction and further computational analyses were carried out to achieve this.

#### 2.3.2 NCI-plot Analysis

NCI-plot analysis is a powerful method to reveal the repulsive or attractive nature of the interaction and to describe the relative strength of noncovalent bonding.^81,82^ 2D and 3D NCI-plots visualizing all the interactions in **1**·I_2_ and **2**·I_2_ cocrystals can be found in Figures S8–S13. Here only the interactions involved in the bifurcated contact are discussed.

**Figure 4:**
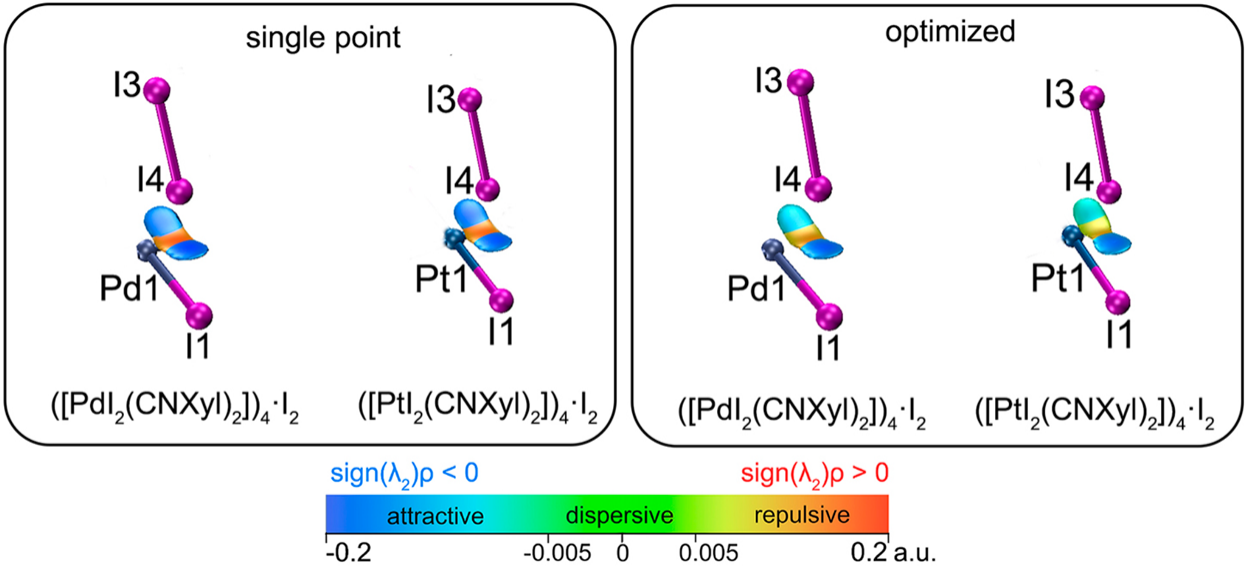
NCI visualizations of the bifurcated contact for SP (left) and OPT (right) *trans*-[MI_2_(CNXyl)_2_]_4_·I_2_ clusters (where M = Pd or Pt). Corresponding 2D graphs, as well as full 3D visualizations containing all the interactions, can be found in Figures S8–S13.

For (**1**)_4_·I_2_ and (**2**)_4_·I_2_ clusters 2D plots of (*s*) against sign(λ_2_)ρ have a similar shape (see Figures S8 and S9). Two types of attractive NCIs (I···I and I···M XBs, where M = Pt or Pd) were found in the [−0.02, −0.008] a.u. range of sign(λ_2_)ρ and one type of repulsive interaction found in the [0.009, 0.018] a.u. range of sign(λ_2_)ρ (Figure 4). The repulsion areas for the I–I···(I–M) interactions can be explained by the repulsion of lone pairs of the metal center and iodide ligand in **1** and **2**; the same areas can be found in isolated **1** and **2** (see the sign(λ_2_)ρ projections in Figure S14). Expectedly, the strength of XB in both cocrystals was found to be very similar. This observation correlates with data obtained experimentally (see Table 1).

Especially intriguing is the difference between SP and OPT structures. As expected, the strength of all interactions weakens in the optimized structures (Figure 4, Table 3). In the case of SP structures, I···I and I···M contacts have very similar interaction strengths, while in the OPT structures I···M contact is weaker. In the case of (**2**)_4_·I_2_, the change is more noticeable (i.e., interactions are more weakened) than that in the case of (**1**)_4_·I_2_ (Table 3).

**Table 3:** Peak sign(λ_2_)ρ Values of NCIs in the (**1**)_4_·I_2_ and (**2**)_4_·I_2_ (a.u.)

cluster	(**1**)_4_·I_2_, M = Pd		(**2**)_4_·I_2_, M = Pt	
	peak sign(λ_2_)ρ, a.u.		peak sign(λ_2_)ρ, a.u.	
interaction	SP	OPT	SP	OPT
I4···I1	–0.0167	–0.0154	–0.0164	–0.0145
I3···I2				
I4···M1	–0.0159	–0.0119	–0.0160	–0.0099
I3···M2				

#### 2.3.3 Philicity Definition: Analysis of ELF and ED/ESP Minima

ELF is useful in the investigation of XBs and related interactions.^24,83−89^ As a derivative of electron density ELF^69,90−92^ allows to locate areas of shared and unshared electron pairs. A combination of ELF and QTAIM^70^ methods visualizes bond paths at the interaction areas and facilitates defining the philicity of interacting atoms.^26,93^

**Figure 5:**
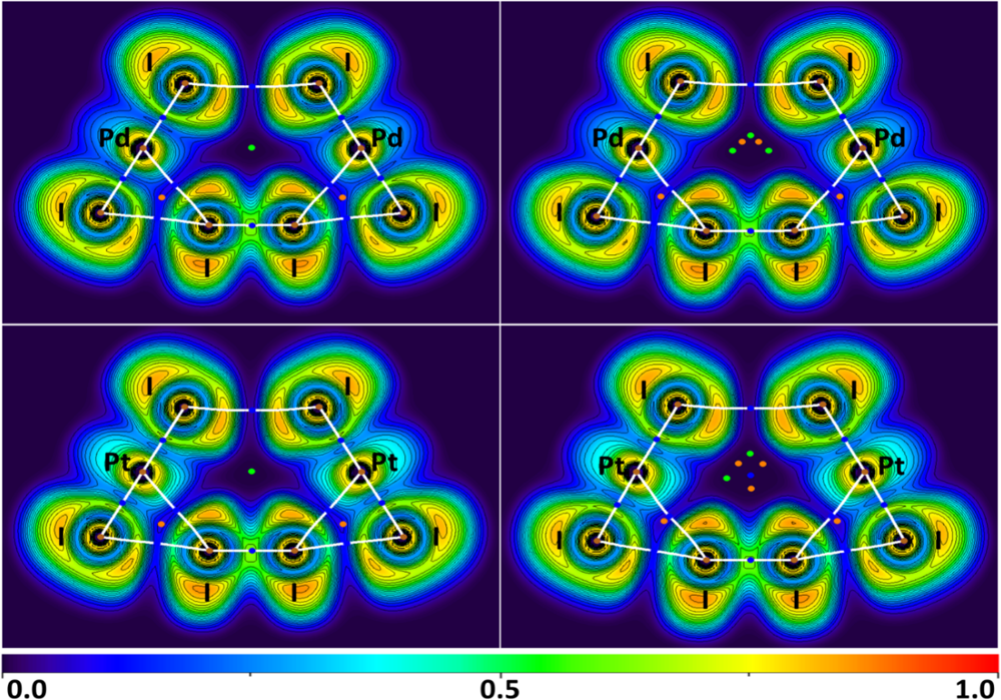
ELF projections with plotted contour lines (black, step is 0.05), bond paths (white lines), BCPs (blue dots), nuclear critical points (NCPs, brown dots), ring critical points (RCPs, orange dots), and cage critical points (green dots) for the I–I···I and I–I···M interactions in the SP (**1**)_4_·I_2_ (upper left), OPT (**1**)_4_·I_2_ (upper right), SP (**2**)_4_·I_2_ (lower left), and OPT (**2**)_4_·I_2_ (lower right) model clusters.

Combined ELF and QTAIM analysis information for SP and OPT (**1**)_4_·I_2_ and (**2**)_4_·I_2_ model structures is presented in Figure 5 as projections on a plane formed by metal atoms, iodide ligands, and iodine molecules. In all four analyzed structures, the I–I···(I–M) bond paths go through the increased ELF areas on the iodides (i.e., through the lone pairs) and through decreased ELF regions on the diiodine I atoms (i.e., through the σ-holes). These observations support the XB nature of the I–I···(I–M) contacts where iodide ligands behave as nucleophiles toward electrophilic diiodine molecules. Similar behavior was observed in the case of the I–I···(I–Pt) XBs in [PtI_2_(1,5-cyclooctadiene)]·0.5I_2_ in our previous work,^26^ where the I···I bond paths go through the σ-hole (iodine in I_2_) and the lone electron pair (iodide ligand) ELF regions.

ELF projections show increased ELF areas around Pd and Pt atoms above and below the bond paths connecting metal centers and iodide ligands that can be interpreted as filled d_*z*^2^_ orbitals. The M···I bond paths that connect metal centers and I_2_ molecules go through these d_*z*^2^_ orbitals. However, the ELF values suggest only relatively weak concentrations of electron pairs in areas occupied by d_*z*^2^_ orbitals and the areas lack directional dependence outside the plane formed by metal centers and iodide ligands suggesting that metal centers are likely to act as weak nucleophiles at most. Actually, any bond path corresponding to NCI with the metal center would cross the area of d_*z*^2^_ orbitals, because any d^8^-metal-involving interaction is required to stay away from the ligands in the complex plane, and the nature of the NCI will depend more on the atom of the interacting partner. At the same time, in all four clusters the I···M bond paths connect to I_2_ iodine atoms through areas with intermediate ELF values which could indicate that the interactions are either weakly polar or nonpolar. Since the I···M bond paths connect atoms in each structure through areas described by intermediate ELF values, combined ELF and QTAIM analysis does not provide conclusive evidence on the philicity of atoms in these interactions.

An alternative way to assign philicity of noncovalently interacting atoms is to compare the minima of the electron density (ED) and ESP along the bond path.^24,87,88,94−97^ In polar NCIs the minimum of ESP is shifted toward the nucleophilic atom, while the ED minimum is shifted toward the electrophilic atom. In the case of the I–I···(I–M) interactions (M = Pd or Pt), 1D profiles of the ED and ESP functions along the I–I···I–M bond paths confirm the iodide nucleophilicity toward diiodine in all four clusters as shown in Figures S15 and S16.

**Figure 6:**
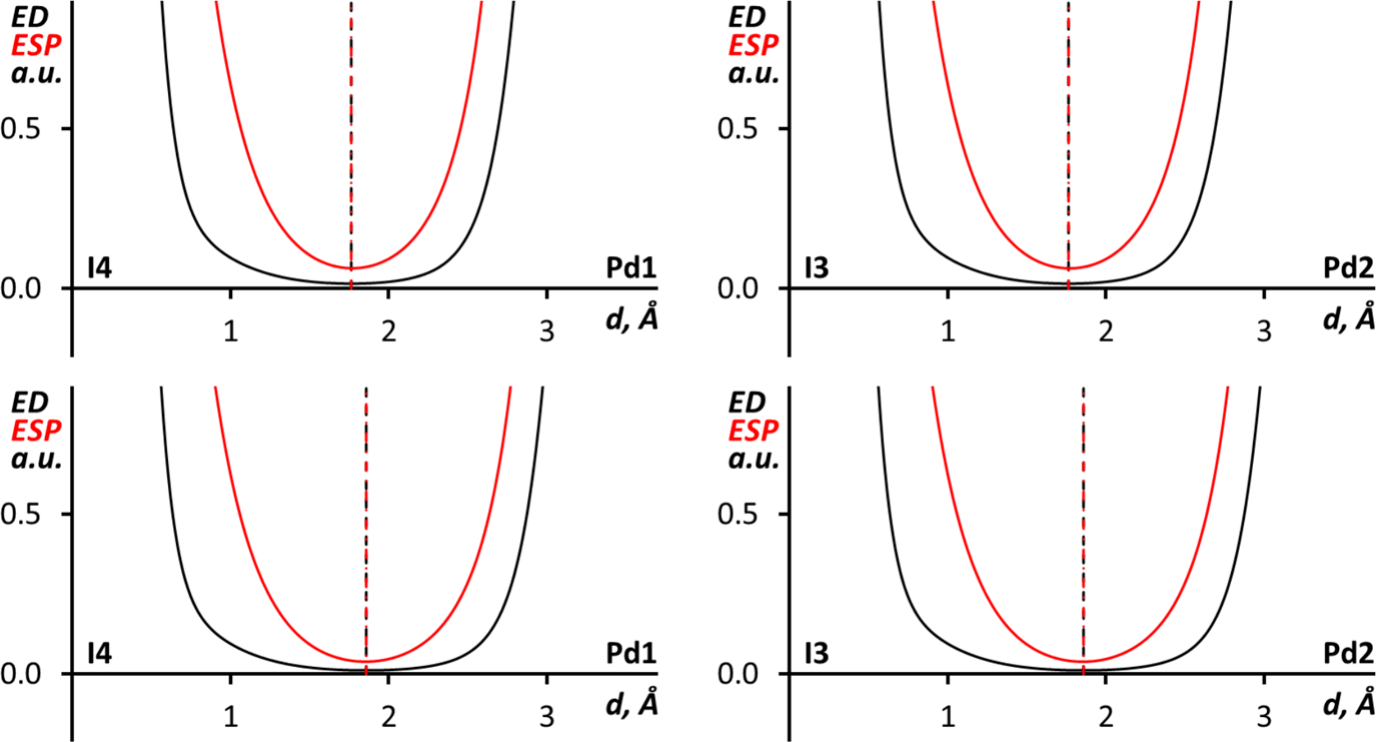
1D profiles of the ED (black) and ESP (red) functions along the I···Pd bond paths in (**1**)_4_·I_2_ for SP (upper graphs) and OPT (lower graphs) structures.

In the case of I···Pd bond paths in (**1**)_4_·I_2_, the 1D profiles of the ED and ESP functions (Figure 6) show that their minima overlap both in the SP and OPT structures. Together with the combined ELF and QTAIM analysis information, this suggests that I···Pd interactions are best described as nonpolar with Pd and I atoms having similar roles. It is noteworthy that similar interactions, which can be also called intermediate between semicoordination (electrophilic metal center) and metal-involving halogen bonding (nucleophilic metal center), have been previously reported for a Ni(II) complex.^31^

**Figure 7:**
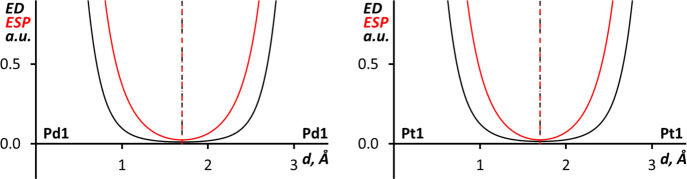
1D profiles of the ED (black) and ESP (red) functions along the Pd···Pd bond path in (*cis*-[PdCl_2_(CNPh)_2_])_2_ (left) and the Pt···Pt bond path in (*cis*-[PtCl_2_(CNPh)_2_])_2_ (right).

The nonpolar noncovalent I···Pd interactions in (**1**)_4_·I_2_ are reminiscent of the noncovalent metal center involving interactions in related palladium and platinum chloride isocyanide complexes,^98−101^ where metal centers participate in nonpolar metallophilic Pd···Pd and Pt···Pt bonds. To compare these interactions DFT SP calculations (M06-L/def2-TZVP) were carried out for two model clusters (*cis*-[PdCl_2_(CNPh)_2_])_2_ and (*cis*-[PtCl_2_(CNPh)_2_])_2_, based on the experimental X-ray data from the structures COYBOI01 and CPICPT12,^99^ respectively. Combined ELF and QTAIM analysis of the (*cis*-[MCl_2_(CNPh)_2_])_2_ (M = Pd or Pt) clusters indicated the expected existence and nonpolar noncovalent nature of the Pd···Pd and Pt···Pt interactions (see Figure S18). Further confirmation of the nonpolar nature of the metallophilic interactions is provided by the 1D profiles of the ED and ESP functions along the M···M bond paths in (*cis*-[MCl_2_(CNPh)_2_])_2_ (M = Pd or Pt) clusters (Figure 7) where ED and ESP minima overlap in both cases.

The ED/Laplacian of ED values in Pd···Pd (0.012/0.030 a.u.) and Pt···Pt (0.016/0.038 a.u.) BCPs in (*cis*-[MCl_2_(CNPh)_2_])_2_ clusters are similar to the values in the I···Pd BCPs in (**1**)_4_·I_2_ SP (0.016/0.037–0.038 a.u.) and (0.012/0.027 a.u.) OPT structures. The nonpolarity of the I···Pd interactions in (**1**)_4_·I_2_ and the similarity of their strength to metallophilic interactions leads us to designate them as *quasimetallophilic* interactions.

**Figure 8:**
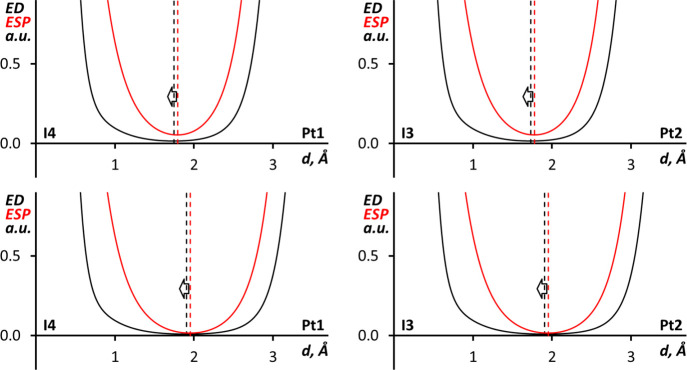
1D profiles of the ED (black) and ESP (red) functions along the I···Pt bond paths in (**2**)_4_·I_2_ SP (upper graphs) and OPT (lower graphs) structures.

Comparison of the 1D profiles of ED and ESP along the I···Pt bond paths in (**2**)_4_·I_2_ SP and OPT structures (Figure 8) shows that the ESP minima are slightly shifted toward the Pt atoms. The shift indicates that Pt atoms act as weak nucleophiles toward I_2_ iodine atoms, and the I–I···Pt can be treated as metal-involving halogen bonding.^22,23,25^ Interestingly the more nucleophilic character of Pt^II^ compared to Pd^II^ in isostructural cocrystals was previously observed for the X_2_CH–X···M (X = Br and I; M = Pd or Pt) halogen bonding.^23^

However, the small values of the shifts between ED and ESP minima and the ∠(I–I···Pt) angles (128.1–128.3° in SP and 126.5° in OPT structures) that are far from linear leave open the possibility of considering the I···Pt interactions as having intermediate^31^ philicity i.e. treating them as nonpolar interactions.

#### 2.3.4 LED Analysis

**Figure 9:**
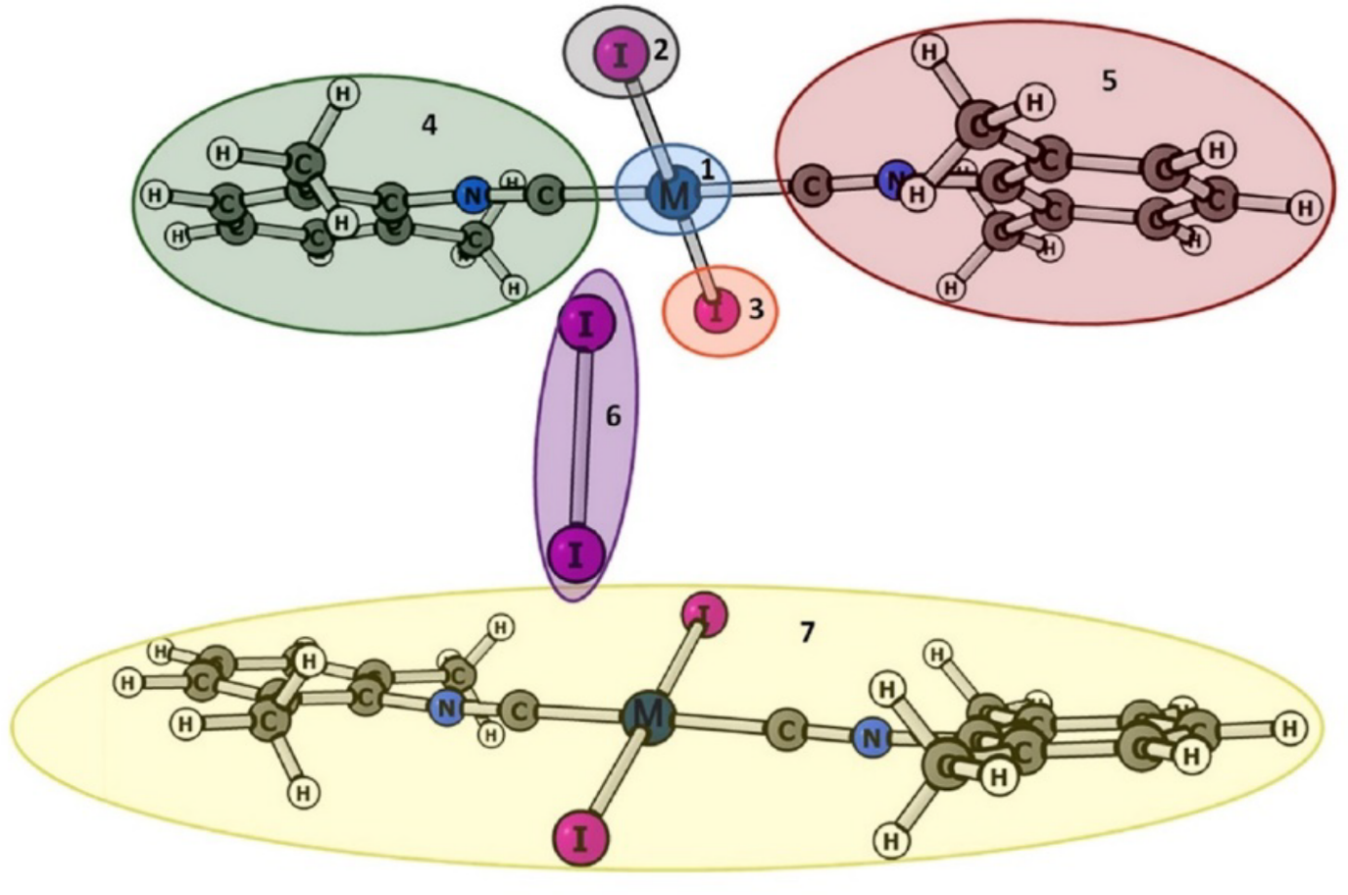
Fragments of (*trans*-[MI_2_(CNXyl)_2_])_2_·I_2_ structures (M = Pd or Pt) used in the local energy decomposition analysis.

The relative energy contributions different types of interactions have to the total interaction can be estimated with local energy decomposition (LED) analysis.^102^ To elucidate the nature of the I···M and I···I interactions, further local energy decomposition (LED) analysis^71^ on DLPNO–CCSD(T)^103−105^/def2-TZVPP^106,107^ wave function for fragments depicted in Figure 9 was carried out. The LED analysis results are given in Table 4. Comparison of the interaction energies between fragments 6 (I_2_) and 7 (*trans*-[MI_2_(CNXyl)_2_], M = Pd or Pt) with energies between fragments 2 (I) and 6 suggest that the interaction between I_2_ and *trans*-[MI_2_(CNXyl)_2_] in both cocrystals of Pd and Pt complexes is almost solely due to the XB between I_2_ and iodide coordinated to M. The interaction between M atom and I_2_ appears to have only a minor supporting role to the total interaction between I_2_ and the complex. This conclusion is in accordance with SCXRD analysis data (based on *R*_IX_ index, I···I XB is slightly stronger than I···M, Table 1). The I···I_2_ XB interaction is classified as mainly electrostatic by the LED analysis with small covalent and dispersion contributions. The weaker I···M interaction has higher contributions from covalent and dispersion terms than does the stronger I···I XB interaction in line with the other analyses that described the I···M interaction as weakly polar or nonpolar.

**Table 4:** Energy Components of the Interfragment Interaction Energies (kJ mol^–1^) in (**1**)_2_·I_2_ and (**2**)_2_·I_2_ Cocrystals Calculated at DLPNO–CCSD(T)/def2-TZVPP Level^a^

cocrystal	interaction	*E*_exch_	*E*_elstat_	*E*_DISP_	*E*_(T)_	*E*_sum_
(**1**)_2_·I_2_	1 ↔ 6	–11	–28	–4	–1	–44
	2 ↔ 6	–64	–254	–11	–4	–333
	6 ↔ 7	–82	–295	–26	–8	–411
(**2**)_2_·I_2_	1 ↔ 6	–10	–27	–5	–1	–43
	2 ↔ 6	–55	–243	–10	–4	–312
	6 ↔ 7	–69	–288	–29	–7	–392

a Exchange interaction, *E*_exch_; electrostatic and polarization energy, *E*_elstat_; dispersion interaction, *E*_DISP_; and contribution from triples correction, *E*_(T)_. Only interactions of interest are represented in this table, detailed information on all the interactions can be found in Supporting Information (Tables S8–S9). Electronic preparation energies resulting from intrafragment changes in electron density and deformation energies due to geometrical differences of fragments in interacting structure compared to their separated equilibrium geometries that are required to derive the dissociation energies corresponding to the analyzed interactions have not been included in the analysis.

## 3 Conclusion

Two novel cocrystals of *trans*-[MI_2_(CNXyl)_2_]·I_2_ (where M = Pd or Pt) representing noncovalently linked metallopolymeric structures were synthesized and characterized. Analysis of crystal structure showed that *trans*-[MI_2_(CNXyl)_2_] units are interlinked via an uncommon I–I···(I–M) bifurcated contact with the I_2_ molecule. Bifurcated contact, in turn, can be subdivided into a I···I halogen bond and a I···M metal-involving interaction. To reveal the nature of the contact, it was studied with various computational methods such as NCI-plot, QTAIM and LED analyses, and ED/ESP minima comparisons. It was shown that the I···I halogen bond is the strongest NCI stabilizing the system, supported by a weaker I···M metal-involving interaction. ED/ESP minima comparisons showed the nonpolarity of I···M contact in the [PdI_2_(CNXyl)_2_]·I_2_ cocrystal; therefore, this interaction was suggested to be called *quasimetallophilic*. In the case of the [PtI_2_(CNXyl)_2_]·I_2_ cocrystal, similar studies showed the weakly nucleophilic nature of Pt center, which makes the I···Pt interaction polar and is best described as metal-involving halogen bonding. However, the differences between the I···Pd and I···Pt interactions are not crucial for directed crystal engineering, and the Pd/Pt isostructural exchange can be further used in the design of similar Pd- and Pt-containing cocrystals.

## 4 Experimental Section

### 4.1 General Computational Details

All the studied structures were optimized and analyzed using DFT theory. To achieve a good compromise between accuracy and computational demand for calculating systems containing NCIs M06-L functional^108^ combined with triple-ζ def2-TZVP^106^ basis sets was chosen as the calculation method. To further reduce computational time resolution of identity approximation^109^ together with def2-TZV density fitting basis sets^106^ was employed in the calculations. DFT calculations were carried out with Gaussian16 (revision C.01) program package.^110^ Complexes **1** and **2** and I_2_ were subjected to full energy minimization. Models for solid-state clusters (**1**)_4_·I_2_ and (**2**)_4_·I_2_ were directly cut from the corresponding experimental crystal structures. Bonding analyses of NCIs in model structures (**1**)_4_·I_2_ and (**2**)_4_·I_2_ were carried out on both optimized (OPT) and crystal structure derived SP structures (where only positions of H-atoms were optimized). SP calculations (M06-L/def2-TZVP) were also carried out for two model clusters, (*cis*-[PdCl_2_(CNPh)_2_])_2_ and (*cis*-[PdCl_2_(CNPh)_2_])_2_, based on the experimental X-ray data from the structures COYBOI01 and CPICPT12,^99^ respectively. The strength and topology of the interactions were studied with the NCI-plot program^68^ implemented in Critic2 software,^111^ and 2D and 3D visualizations were carried out in Gnuplot^112^ and VMD programs^113^ respectively. ESP surfaces of **1**, **2**, and I_2_ molecules were calculated and visualized using AIMALL software^114^ at 0.001 a.u. surfaces. ELF projections and QTAIM analyses were carried out in Multiwfn 3.7.^115^ DLPNO–CCSD(T)^103−105^ wave functions for the LED analyses,^71^ and the analyses themselves were calculated with ORCA 4.2 program^116^ using def2-TZVPP^106^ orbital and def2-TZVPP/C^107^ and def2/JK^117^ auxiliary basis sets.

### 4.2 Materials and SCXRD Details

All chemicals and solvents such as CHCl_3_ (VWR BDH Chemicals), CH_2_Cl_2_ (VWR BDH Chemicals), acetone (Fisher Scientific), KI (≥99.0%, Fisher Scientific), I_2_ (Mallinckrodt), 2,6-dimethylphenyl isocyanide (further CNXyl, ≥98.0 GC%, Aldrich), and [PdCl_2_(CH_3_CN)_2_] (99%, Aldrich) were used without additional purification. [PtI_2_COD] was synthesized according to the procedure reported by Rigamonti et al.^61^ The crystal data and details of data processing for the obtained cocrystals are summarized in the Supporting Information (“Single Crystal X-ray Diffraction data analysis (SCXRD)” section).

***Caution**! CNXyl is hazardous to health and should be handled with care.*

#### 4.2.1 Synthesis of *trans*-[PdI_2_(CNXyl)_2_]

Synthesis was adapted from a procedure presented by Crociani et al.^58^ Solid CNXyl (26.2 mg, 0.2 mmol) was added to the suspension of [PdCl_2_(CH_3_CN)_2_] (25.9 mg, 0.1 mmol) in 5 mL of CHCl_3_. The reaction mixture was refluxed with stirring for 3 h and then cooled to room temperature (RT), and the solvent was evaporated at a rotary evaporator to give *cis*-[PdCl_2_(CNXyl)_2_] as a white solid. Then solid KI (166 mg, 1 mmol) was added to *cis*-[PdCl_2_(CNXyl)_2_] (43.7 mg, 0.10 mmol), and acetone (20 mL) was added to the resulting mixture. The resultant yellow suspension was stirred at RT for 2 days. The solvent was then fully evaporated on a rotary evaporator at 50 °C, and the orange product was suspended in H_2_O. The product was extracted with CH_2_Cl_2_. The organic fraction was subjected to full solvent evaporation on a rotary evaporator, and the resulted orange solid was dissolved in CHCl_3_. Some white insoluble material was filtered off from solution; the filtrate was left for recrystallization at RT in darkness (from CHCl_3_). The yield of orange crystalline product was 55.6 mg (0.09 mmol, 89%). Elemental analysis (EA) CHN mode: Found: C 35.72; H 3.22; N 4.44. Calcd: C 34.73; H 2.91; N 4.50. ^1^H NMR (300 MHz, CDCl_3_, δ ppm): 2.55 (s, 12H), 7.09–7.14 (m, 4 H), 7.21–7.28 (m, 2H).

#### 4.2.2 Synthesis of I_2_ Cocrystal of *trans*-[PdI_2_(CNXyl)_2_]

*trans*-[PdI_2_(CNXyl)_2_] (24.9 mg, 0.04 mmol) and I_2_ (15.2 mg, 0.06 mmol) were dissolved in CH_2_Cl_2_/CHCl_3_ (50:50 mixture, 8 mL). The solution was stirred at 50 °C (to dissolve iodine fully) until the mixture became homogeneous and then left for crystallization in dark at RT. The phase purity of the bulk material was confirmed by powder X-ray diffraction (PXRD, see the Supporting Information).

#### 4.2.3 Synthesis of *trans*-[PtI_2_(CNXyl)_2_]

Synthesis was adapted from a procedure presented by Kaharu et al.^59^ [PtI_2_COD] (83 mg, 0.15 mmol) was added to a 5 mL of CH_2_Cl_2_ solution of a CNXyl (39.4 mg, 0.3 mmol), and the mixture was stirred for 3 days at RT in darkness. The solvent was evaporated, and obtained solid was crystallized from CH_2_Cl_2_. TLC (silica gel 60 plate + CHCl_3_) revealed byproducts. The product was purified by column chromatography (silica gel 60 + CHCl_3_) and recrystallized from CHCl_3_. The yield of yellow crystalline product was 99.8 mg (0.14 mmol, 93%). EA CHN mode: Found: C 31.83; H 2.81; N 4.11. Calcd: C 30.40; H 2.55; N 3.94. ^1^H NMR (300 MHz, CDCl_3_, δ ppm): 2.58 (s, 12H), 7.14–7.18 (m, 4 H), 7.26–7.32 (m, 2H).

#### 4.2.4 Synthesis of I_2_ Cocrystal of *trans*-[PtI_2_(CNXyl)_2_]

*trans*-[PtI_2_(CNXyl)_2_] (21.3 mg, 0.03 mmol) and I_2_ (15.2 mg, 0.06 mmol) were dissolved in CHCl_3_, and the resulting dark brown mixture was left in an aluminum foil covered vial for slow evaporation at ambient conditions to give dark brown crystals of the desired product. The phase purity of the bulk material was confirmed by PXRD analysis (see Supporting Information).

## Supporting Information Available

Single-crystal X-ray diffraction data analysis (experimental procedures and crystallographic details); PXRD of **1**·I_2_ and **2**·I_2_ cocrystals (experimental procedures and detailed results of PXRD analysis); summary of computational studies on NCIs in **1**·I_2_ and **2**·I_2_ cocrystals (general computational details, ESP, QTAIM, LED, and NCI-plot analyses, ED/ESP minima criterion for I···I interactions); view of (*cis*-[MCl_2_(CNPh)_2_])_2_ (M = Pd or Pt) dimeric clusters and ELF projections for them (PDF)

## Accession Codes

CCDC 2054859–2054862 contain the supplementary crystallographic data for this paper. These data can be obtained free of charge via www.ccdc.cam.ac.uk/data_request/cif, or by emailing data_request@ccdc.cam.ac.uk, or by contacting The Cambridge Crystallographic Data Centre, 12 Union Road, Cambridge CB2 1EZ, UK; fax: +44 1223 336033.
