# Supplementary material for: Studies of Nature of Uncommon Bifurcated I–I···(I–M) Metal-Involving Noncovalent Interaction in Palladium(II) and Platinum(II) Isocyanide Cocrystals
Source: Inorg Chem. 2021 Aug 6;60(17):13200–11. doi: 10.1021/acs.inorgchem.1c01591 (PMC8424624; doi:10.1021/acs.inorgchem.1c01591)
Supplement: Supplementary file 1 — ic1c01591_si_001.pdf [file ic1c01591_si_001.pdf]

Supporting Information

Studies of Nature of Uncommon Bifurcated I–I···(I–M) Metal-involving Noncovalent Interaction in Palladium(II) and Platinum(II) Isocyanide Cocrystals

Margarita Bulatova[a], Daniil M. Ivanov[b], J. Mikko Rautiainen[a], Mikhail A. Kinzhalov[b], Khai-Nghi Truong[a], Manu Lahtinen[a], Matti Haukka[a,*]

[a]Department of Chemistry, University of Jyväskylä, P.O. Box 35, FI-40014, Jyväskylä, Finland, e-mail: matti.o.haukka@jyu.fi

[b]Institute of Chemistry, Saint Petersburg State University, Universitetskaya Nab. 7/9, Saint Petersburg, 199034 Russian Federation, e-mail: d.m.ivanov@spbu.ru

Table of Contents

Supporting Information S1

Numbering of Complexes S3

Single crystal X-ray Diffraction data analysis (SCXRD) S3

Experimental Procedures S3

Crystallographic Details S4

Powder X-ray diffraction analysis (PXRD) of 1·I_2 and 2·I_2 cocrystals S5

Experimental Procedures S5

Detailed results of PXRD analysis S6

Summary of computational studies on noncovalent interactions in 1·I_2 and 2·I_2 cocrystals S9

General Computational details S9

ESP analysis S9

QTAIM analysis S11

LED analysis S14

NCI-plot analysis. Experimental Procedures S15

NCI-plot analysis. Results S15

Analysis of electron localization function (ELF) and ED/ESP minima S22

References S24

Numbering of Complexes

Table S1. Numbering of Complexes.

Complex	Number
trans-[$PdI_2(CNXyl)_2$]	**1**
trans-[$PtI_2(CNXyl)_2$]	**2**

Single crystal X-ray Diffraction data analysis (SCXRD)

Experimental Procedures

For each experiment, single crystals were selected from the sample under microscope, immersed in cryo-oil, and mounted in a MiTeGen loop for the SCXRD data collection. All data were measured using a dual-source Rigaku SuperNova diffractometer equipped with an Atlas detector and an Oxford Cryostream cooling system using mirror-monochromated Mo-K_α radiation (λ = 0.71073 Å). Data collection and reduction for all complexes were performed using the program CrysAlisPro[1] and Gaussian face-index absorption correction method was applied.[1] All structures were solved with Direct Methods or Patterson synthesis (SHELXS)[2] and refined by full-matrix least squares based on F^2 using SHELXL-2013.[3] Anisotropic displacement parameters were assigned for all non-hydrogen atoms unless stated otherwise. In the structure **1**·I_2 there is a short contact between I1 and I4 atoms because of the halogen bonding between these two iodines. There is also a similar interaction between I3 and I2. Hydrogen atoms were placed in their idealized positions and refined as riding atoms. Isotropic displacement parameters for all H atoms were constrained to multiples of the equivalent displacement parameters of their parent atoms with U_{iso}(H) = 1.2 U_{eq}(parent atom). Enhanced rigid bond restraints (RIGU)[4,5] with standard uncertainties of 0.001 Å[6] were applied for several atom pairs. The single crystal X-ray data, experimental details and CCDC numbers (2054859-2054862) are given below.

Crystallographic Details

Table S2. Crystal data for **1**, **2**, **1**·I_2, and **2**·I_2

**Cocrystal**	**1**	**2**	**1**·I_2	**2**·I_2
**CCDC No:**	2054861	2054860	2054859	2054862
Empirical formula	$C_{18}H_{18}I_2N_2Pd$	$C_{18}H_{18}I_2N_2Pt$	$C_{18}H_{18}I_4N_2Pd$	$C_{18}H_{18}I_4N_2Pt$
Formula weight	622.54	711.23	876.34	965.02
Temperature (K)	170(2)	171(2)	120(2)	100(2)
λ(Å)	0.71073	0.71073	0.71073	0.71073
Crystal system	monoclinic	monoclinic	triclinic	triclinic
space group (No.)	$P2_1/c$ (14)	$P2_1/c$ (14)	P-1 (2)	P-1 (2)
a (Å)	8.7303(2)	8.7259(3)	8.5002(4)	8.5273(2)
b (Å)	16.8756(5)	16.8556(4)	10.6606(6)	10.6177(3)
c (Å)	7.0916(2)	7.0885(2)	13.9757(9)	14.0149(5)
α (°)	90	90	70.934(2)	70.758(2)
β (°)	108.3330(10)	108.5100(10)	90.006(3)	89.960(3)
γ (°)	90	90	72.260(2)	72.496(2)
V (Å^3)	991.77(5)	988.64(5)	1133.04(11)	1135.71(6)
Z	2	2	2	2
ρ_{calc} (g/cm^3)	2.085	2.389	2.569	2.822
μ(Kα) (mm^{-1})	4.049	10.220	6.273	11.619
Crystal size/mm^3	0.117 × 0.087 × 0.058	0.173 × 0.078 × 0.049	0.124 × 0.038 × 0.015	0.451 × 0.123 × 0.063
2θ range for data collection (°)	4.828 to 55.178	4.834 to 54.956	3.102 to 50.496	6.094 to 50.498
Reflections collected	14809	22926	4117	4113
Independent reflections	2287	2255	4117	4113
Goodness-of-fit (F^2)	1.168	1.073	1.021	1.015
R_1[a] ($I \geq 2\sigma$)	0.0416	0.0296	0.0387	0.0278
wR_2[b] ($I \geq 2\sigma$)	0.0645	0.0621	0.0749	0.0326
$\Delta\rho_{max}/\Delta\rho_{min}$ (e Å^{-3})	0.63/-0.59	1.65/-1.68	2.24/-1.85	1.31/-1.50

[a] $R_1 = \Sigma||F_o| - |F_c||/\Sigma|F_o|$; [b] $wR_2 = [\Sigma[w(F_o^2 - F_c^2)^2]/ \Sigma[w(F_o^2)^2]]^{1/2}$.

Powder X-ray diffraction analysis (PXRD) of 1·I_2 and 2·I_2 cocrystals

Experimental Procedures

Powder X-ray diffraction measurements were done using PANalytical X´Pert PRO diffractometer in Bragg–Brentano geometry with Cu-K_α radiation (λ = 1.5419 Å; 45kV, 40mA). A lightly hand-ground powder sample was prepared on a silicon-made “zero–background inducing” holder with shallow sample cavity (petrolatum jelly was used as fastener). Diffraction patterns were recorded from a spinning sample by position-sensitive X´Celerator detector using continuous scanning mode in 2θ range of 6 – 60° with a step size of 0.017° and counting time of 200 s per step. Diffraction data analyses were made using program Malvern Panalytical HighScore Plus (v. 4.8).[7] The room temperature unit cell parameters of **1**·I_2 and **2**·I_2 cocrystals were indexed by Pawley analysis[8] using the corresponding low temperature single crystal structure parameters (presented in this study) of both phases as the basis of least-squares refinement. Parameters varied in the whole-pattern fitting were as follows: zero-offset, polynomial background, sample displacement, unit cell and peak profile parameters.

Detailed results of PXRD analysis

According to the obtained results (Figure S1–Figure S2,Table S3) phase purity of the bulk material was confirmed for both **1**·I_2 and **2**·I_2 cocrystals.

Table S3. Crystallographic data of complexes **1**·I_2 and **2**·I_2 measured by SCXRD and PXRD.

Parameters	**1**·I_2		**2**·I_2	
	PXRD	SCXRD	PXRD	SCXRD
Temp [°C]	22	-153	22	-173
Crystal system	triclinic	triclinic	triclinic	triclinic
Space group	*P*-1	*P*-1	*P*-1	*P*-1
a /Å	8.557(2)	8.5002(4)	8.564(1)	8.5273(2)
b /Å	10.765(2)	10.6606(6)	10.730(1)	10.6177(3)
c /Å	14.215(3)	13.9757(9)	14.246(2)	14.0149(5)
α /°	70.558(3)	70.934(2)	70.468(2)	70.758(2)
β /°	89.932(2)	90.006(3)	89.657(1)	89.960(3)
γ /°	72.313(3)	72.260(2)	72.473(2)	72.496(2)
V/Å^3	1169.14	1133.04(11)	1169.99	1135.71(6)
$R_{exp.}$	0.0298		0.0185	
$R_{prof.}$	0.0372		0.0306	
$R_{w\text{-}prof}$	0.0450		0.0375	
R_1		0.0387		0.0278
wR_2		0.0749		0.0326
GOF	1.513	1.021	2.027	1.015

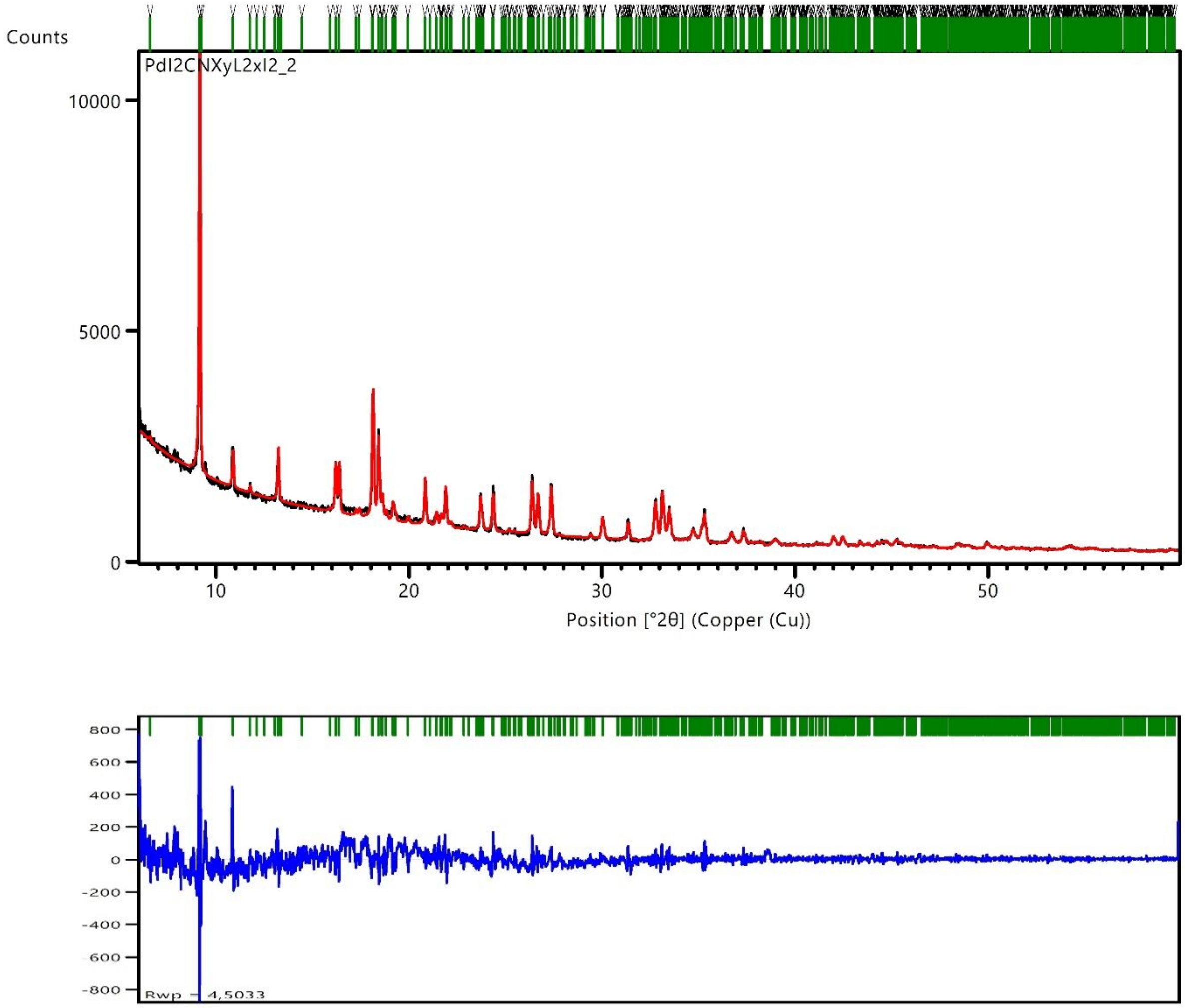

Figure S1**.** Pawley refinement plot of **1**·I_2. Experimental pattern is shown in black and refined profile in red, whereas green colored ticks correspond to Bragg peak positions of the refined unit cell. Difference plot of experimental vs. refined profile is shown below in blue.

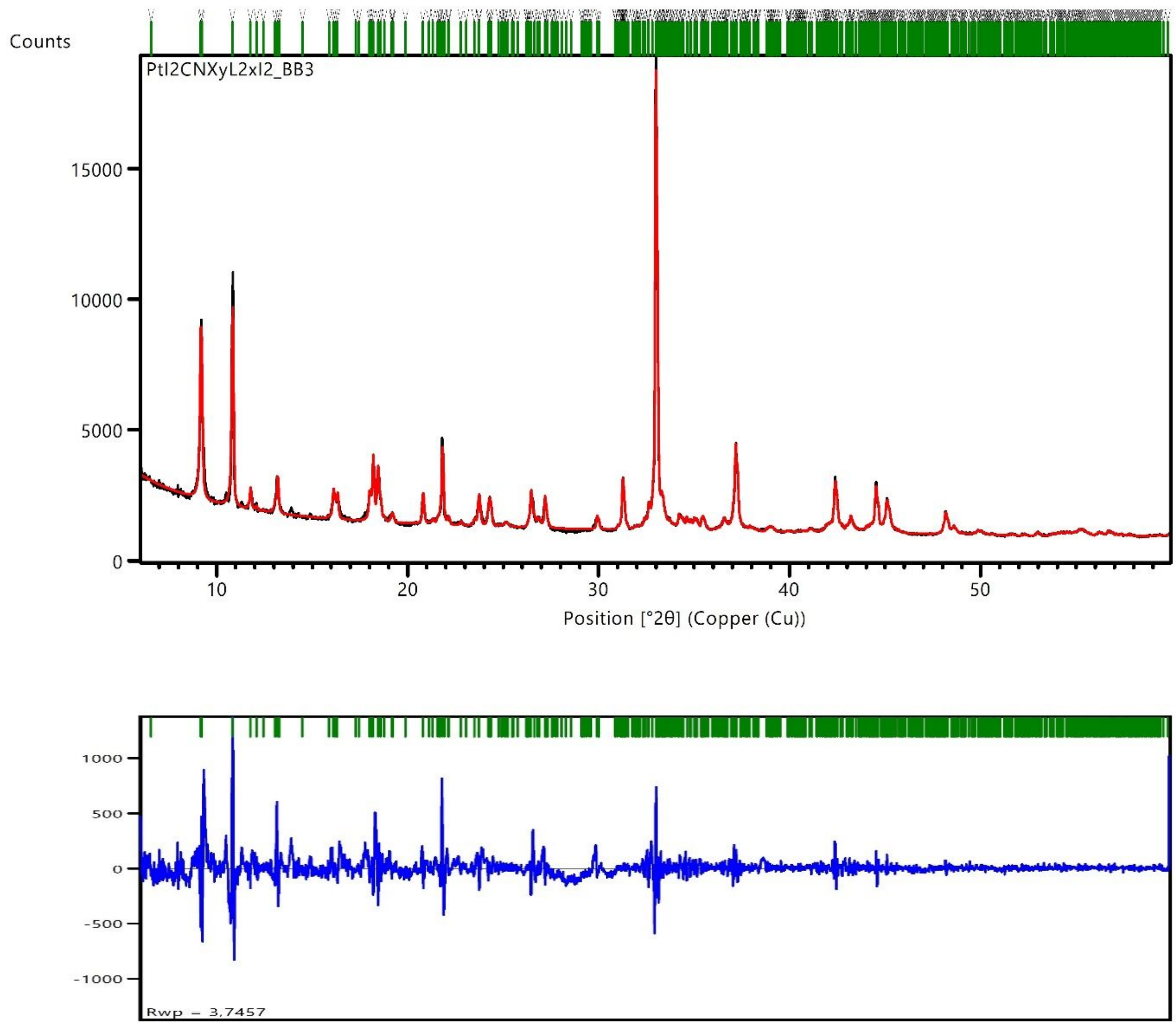

Figure S2**.** Pawley refinement plot of **2**·I_2. Experimental pattern is shown in black, refined profile in red, whereas green colored ticks correspond to Bragg peak positions of the refined unit cell. Difference plot of experimental vs. refined profile is shown below in blue.

Summary of computational studies on noncovalent interactions in $1 \cdot I_2$ and $2 \cdot I_2$ cocrystals

General Computational details

All models were calculated with the Gaussian09 (revision C.01) program package[9] at the DFT level of theory. M06-L[10] density functional was utilized together with the def2-TZVP[11–13] basis set. Strength, topology and nature of noncovalent interactions in the cocrystals were studied using the wave functions obtained from the DFT calculations. Strength and topology of the interactions were studied with NCI-plot program[14] implemented in Critic2 software[15], ESP surfaces of the **1**, **2**, and I_2 molecules were calculated with AIMALL[16] software, nature of the interaction was studied with ESP and ELF analysis implemented in MultiWFN[17] software. Models of **1**, **2**, I_2, $\mathbf{1} \cdot I_2$, and $\mathbf{2} \cdot I_2$ were subjected to full energy minimization. MEPs were calculated in AIMALL software the 0.001 a.u. of the molecules of interest. The models for the solid state structures were directly cut from the corresponding experimental crystal structures creating clusters: $(\mathbf{1})_4 \cdot I_2$ and $(\mathbf{2})_4 \cdot I_2$ (Figure S3). The clusters were analyzed both with and without geometry optimization.

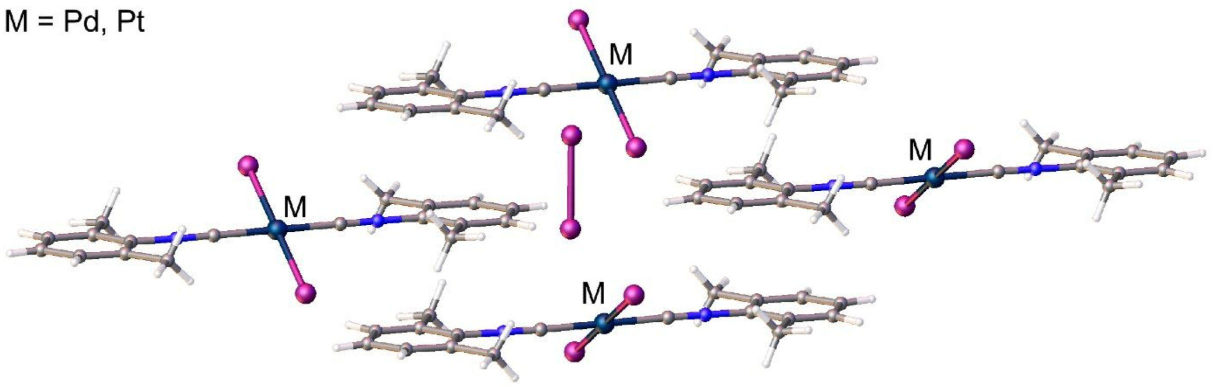

Figure S3. View of $(\mathbf{1})_4 \cdot I_2$ and $(\mathbf{2})_4 \cdot I_2$ model clusters.

ESP analysis

All ESP calculations were done in AIMALL program at 0.001 a.u. contour of molecular surface, utilizing wavefunctions generated at the M06-L/def2-TZVP level of theory.

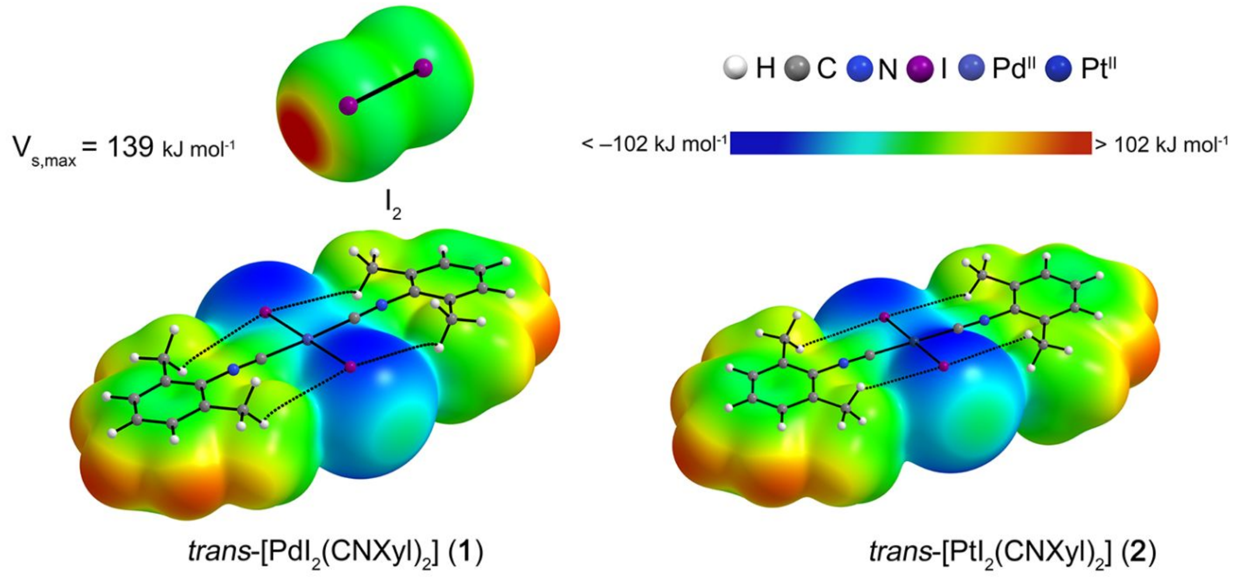

Figure S4. Side view on electrostatic potential calculated at the M06L/def2TZVP/def2TZV computational level on the 0.001 a.u. molecular surface of I_2, **1** and **2** using the same color scale from –102 to 102 kJ mol^{-1}.

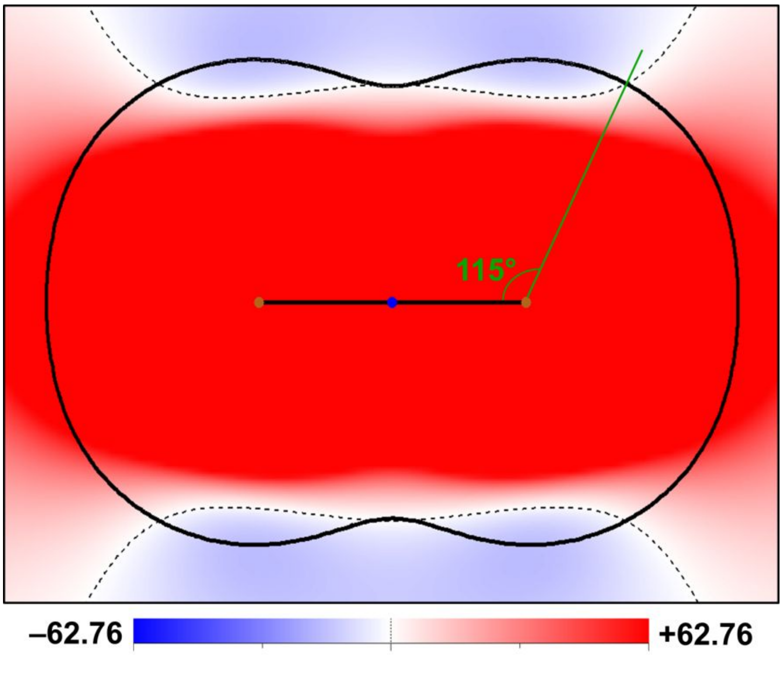

Figure S5. MEP of I_2 molecule (units are kJ·mol^{-1}), where zero ESP is represented by dashed line. The σ-hole limit angle (shown in green in the figure) is a parameter that helps to estimate the area range where the electron-rich atom can approach the electron deficient area of I atom with favorable electrostatic attraction. In case of I_2 this angle is 115−180°.

QTAIM analysis

All attempts to optimize a structure corresponding to the solid-state structure of $(\mathbf{1})_2{\cdot}I_2$ (or $(\mathbf{2})_2{\cdot}I_2$) by using only the components of the simplest structural unit resulted in twisted geometries that retained the interaction between I_2 and **1** (or **2**) but made the two **1** (or **2**) units move almost perpendicular to each other. To retain the structural motif observed in the solid-state, two additional **1** (or **2**) units that take part in the π-π stack formation between Xyl rings had to be included in the optimization as shown in Figure S6. This indicates that π-π interaction between Xyl rings locks the complexes together in the solid-state and directs the crystal structure formation.

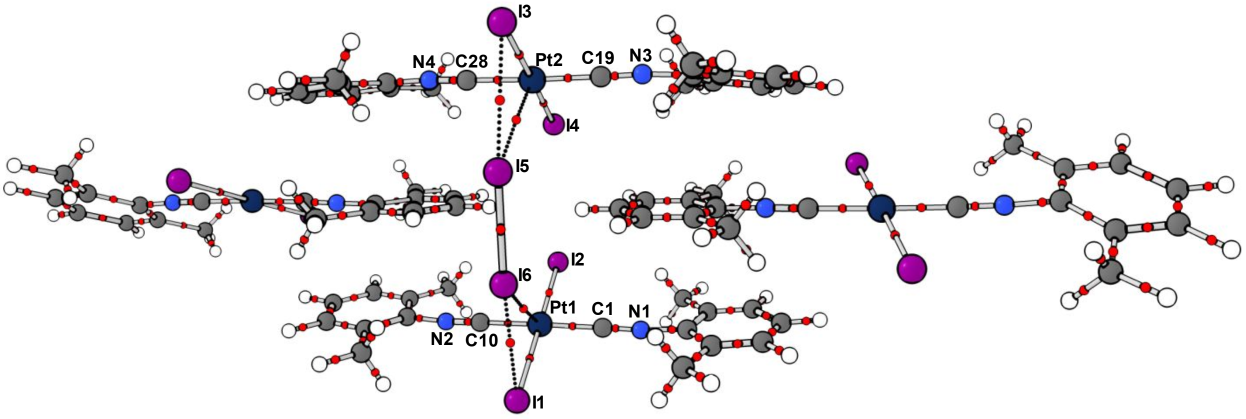

Figure S6. M06-L/def2-TZVP optimized structure of $(\mathbf{2})_2{\cdot}I_2$ with two supporting **2** complexes.

QTAIM parameters calculated for the bonds around the metal centers in the optimized structures (OPT) $(\mathbf{1})_2$ I_2 (and $(\mathbf{2})_2$ I_2) shown in Table C1 (and C2) exhibit electron densities ρ_b and Laplacian values $\nabla^2\rho$ normal for Pt (and Pd) complexes.[18–20] Trends in ρ_b follow the changes in bond distances and all $\nabla^2\rho$ are positive. The positive $\nabla^2\rho$ are observed even for the formal triple bonds between C and N atoms of the CNXyl ligands. This is consistent with the positive $\nabla^2\rho$ values that were observed for polar multiple bonds in HCN and CO already by Cremer and Kraka in 1984[21], or in case of carbonyl ligands in the N-Heterocyclic Carbene Triruthenium Cluster studied by Cabeza et.al.[22] In such systems bond polarity degree influences the electron density Laplacian at BCP. In case of polar bond, it the atomic basin of the more electronegative element dominates, and the interatomic surface is shifted toward the more electropositive atom. Thus, BCP is lying close to the nodal surface of the Laplacian with both V valence shell charge concentrations belonging to the more electronegative basin. The $|V_b|/G_b$ quotient

calculated for C≡N bond (average 1.91 both **1** and **2**) is very close to that found for HCN (1.79).[21]

In addition to describing the strong covalent bonds, the QTAIM analysis also reveals weak interactions from I_2 atoms to both metal center and iodo ligand. In the optimized structures the contact between iodine atoms I1 and I6 is calculated to be stronger than the interaction of metal center and I6, whereas in the crystal structures the contacts are more alike. Calculated atomic charges (See Table C3) suggest that I_2 in $(\mathbf{1})_2$ I_2 (and $(\mathbf{2})_2$ I_2) gains a weak negative charge. Furthermore, the atomic charges indicate that the origin of the transferred electron density are the iodo substituents of **1** (or **2**) complex rather than the metal center. This can be further seen as a sign that halogen bonding between neutral iodine as donor and negatively charged iodo substituents as acceptors is the driving force for the attachment of I_2 to $(\mathbf{1})_2$ I_2 (and $(\mathbf{2})_2$ I_2) structure while the M1-I6 interaction has more of a supporting role in the structure.

Table S4**.** Comparison of selected experimental (SP) and M06-L/def2-TZVP optimized (OPT) structural parameters [Å] of $(\mathbf{2})_2 \cdot I_2$ and their QTAIM bond critical point parameters (in a.u.): electron density ρ_b, Laplacian of electron density $\nabla^2\rho$, local electronic potential energy density V_b, local electronic kinetic energy density G_b.

Contact	Distance		ρ_b		$\nabla^2\rho$		V_b		G_b	
	SP	OPT	SP	OPT	SP	OPT	SP	OPT	SP	OPT
Pt1-I1	2.6177(6)	2.696	0.077	0.067	+0.076	+0.069	–0.068	-0.054	+0.044	+0.035
Pt1-I2	2.6177(6)	2.679	0.077	0.070	+0.068	+0.064	–0.067	–0.055	+0.042	+0.036
Pt1-C1	1.950(7)	1.958	0.157	0.151	+0.413	+0.400	-0.272	–0.257	+0.187	+0.178
Pt1-C10	1.950(7)	1.958	0.157	0.151	+0.413	+0.401	-0.272	–0.257	+0.187	+0.178
Pt2-I3	2.6185(6)	2.695	0.077	0.068	+0.076	+0.069	–0.068	–0.054	+0.044	+0.036
Pt2-I4	2.6185(6)	2.680	0.077	0.070	+0.069	+0.064	–0.067	–0.055	+0.042	+0.036
Pt2-C19	1.939(6)	1.958	0.153	0.151	+0.406	+0.400	-0.263	–0.257	+0.182	+0.178
Pt2-C28	1.939(6)	1.958	0.153	0.151	+0.406	+0.401	-0.263	–0.257	+0.182	+0.178
Pt1-I6	3.4600(6)	3.790	0.016	0.010	+0.037	+0.023	–0.009	–0.005	+0.009	+0.005
Pt2-I5	3.4649(6)	3.789	0.016	0.010	+0.038	+0.023	–0.009	–0.005	+0.009	+0.005
I1-I6	3.5207(8)	3.580	0.016	0.015	+0.034	+0.030	–0.008	–0.006	+0.008	+0.007
I3-I5	3.5194(8)	3.582	0.016	0.015	+0.034	+0.030	–0.008	–0.006	+0.008	+0.007
I2-I4	4.3133(9)	4.637	0.005	0.003	+0.010	+0.007	–0.002	–0.001	+0.002	+0.001
I5-I6	2.7265(8)	2.814	0.070	0.060	+0.021	+0.027	–0.046	–0.035	+0.026	+0.021
C1-N1	1.142(9)	1.157	0.462	0.461	+0.319	+0.320	–1.828	–1.819	+0.954	+0.949
C10-N2	1.142(9)	1.157	0.462	0.461	+0.319	+0.316	–1.828	–1.818	+0.954	+0.948
C19-N3	1.156(8)	1.157	0.477	0.461	+0.424	+0.320	–1.925	–1.818	+1.015	+0.948
C28-N4	1.156(8)	1.157	0.477	0.461	+0.424	+0.316	–1.925	–1.818	+1.015	+0.949

Table S5. Comparison of selected experimental (SP) and M06-L/def2-TZVP optimized (OPT) structural parameters [Å] of $(\mathbf{1})_2{\cdot}I_2$ and their QTAIM bond critical point parameters (in a.u.): electron density ρ_b, Laplacian of electron density $\nabla^2\rho$, local electronic potential energy density V_b, local electronic kinetic energy density G_b.

Contact	Distance		ρ_b		$\nabla^2\rho$		V_b		G_b	
	SP	OPT	SP	OPT	SP	OPT	SP	OPT	SP	OPT
Pd1-I1	2.616	2.705	0.066	0.056	+0.107	+0.091	–0.058	–0.045	+0.042	+0.034
Pd1-I2	2.616	2.675	0.066	0.059	+0.100	+0.089	–0.056	–0.047	+0.041	+0.035
Pd1-C1	1.950	1.967	0.137	0.132	+0.440	+0.428	–0.224	–0.211	+0.167	+0.159
Pd1-C10	1.950	1.967	0.137	0.132	+0.440	+0.428	–0.224	–0.211	+0.167	+0.159
Pd2-I3	2.616	2.705	0.066	0.056	+0.107	+0.091	–0.058	–0.045	+0.042	+0.034
Pd2-I4	2.616	2.675	0.066	0.059	+0.100	+0.089	–0.056	–0.047	+0.041	+0.035
Pd2-C19	1.965	1.967	0.133	0.132	+0.430	+0.428	–0.213	–0.211	+0.160	+0.159
Pd2-C28	1.965	1.967	0.133	0.132	+0.430	+0.428	–0.213	–0.211	+0.160	+0.159
Pd1-I6	3.404	3.590	0.016	0.012	+0.037	+0.027	–0.009	–0.006	+0.009	+0.006
Pd2-I5	3.404	3.591	0.016	0.012	+0.037	+0.027	–0.009	–0.006	+0.009	+0.006
I1-I6	3.499	3.551	0.017	0.015	+0.035	+0.032	–0.008	–0.007	+0.008	+0.007
I3-I5	3.503	3.553	0.017	0.015	+0.035	+0.031	–0.008	–0.007	+0.008	+0.007
I2-I4	4.285	4.481	0.005	0.004	+0.011	+0.008	–0.002	–0.001	+0.002	+0.002
I5-I6	2.740	2.826	0.068	0.059	+0.022	+0.028	–0.044	–0.034	+0.025	+0.021
C1-N1	1.144	1.157	0.476	0.462	+0.386	+0.319	–1.918	–1.829	+1.007	+0.955
C10-N2	1.144	1.157	0.476	0.462	+0.389	+0.322	–1.918	–1.830	+1.007	+0.955
C19-N3	1.148	1.157	0.471	0.462	+0.371	+0.318	–1.886	–1.830	+0.990	+0.955
C28-N4	1.148	1.157	0.471	0.462	+0.373	+0.322	–1.887	–1.829	+0.990	+0.955

Table S6. QTAIM Atomic charges of selected atoms in optimized (OPT) structures of $(\mathbf{1})_2{\cdot}I_2$, $(\mathbf{2})_2{\cdot}I_2$, **1**, and **2**.

Atom	**1**	$(\mathbf{1})_2{\cdot}I_2$	**2**	$(\mathbf{2})_2{\cdot}I_2$	I_2
M1	+0.509	+0.519	+0.441	+0.444	
I1	–0.354	–0.292	–0.313	–0.251	
I2	–0.354	–0.313	–0.313	–0.276	
C1	+0.959	+0.958	+0.949	+0.948	
C10	+0.959	+0.958	+0.949	+0.947	
I6		–0.088		–0.085	0

Table S7. Comparison of structural [Å] and QTAIM bond critical point parameters (in a.u.) of I–I bonds in experimental (SP) and M06-L/def2-TZVP optimized (OPT) structures of $(\mathbf{2})_2{\cdot}I_2$ and I_2: electron density ρ_b, Laplacian of electron density $\nabla^2\rho$, local electronic potential energy density V_b, local electronic kinetic energy density G_b.

	Distance,	ρ_b	$\nabla^2\rho$	V_b	G_b
I5–I6 OPT	2.814	0.060	+0.027	–0.034	0.021
I5–I6 SP	2.7265(8)	0.070	+0.019	–0.044	0.025
I_2	2.695	0074	+0.008	–0.047	0.025

LED analysis

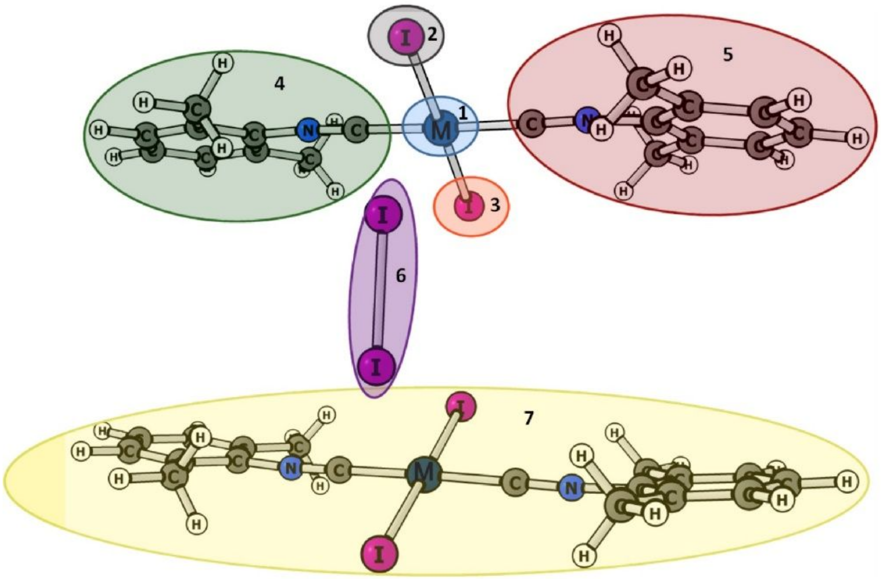

Figure S7. Fragments of $(\mathbf{1})_2\cdot I_2$ (and $(\mathbf{2})_2\cdot I_2$) structure used in the local energy decomposition analysis.

Table S8. Energy components (exchange interaction, E_{exch}, electrostatic and polarization energy, E_{elstat}, dispersion interaction, E_{DISP} and contribution from triples correction, $E_{(T)}$) of the inter-fragment interaction energies (kJ mol^{-1}) in $(\mathbf{2})_2\cdot I_2$ structure calculated at DLPNO-CCSD(T)/def2-TZVPP level.[a]

Interaction	E_{exch}	E_{elstat}	E_{DISP}	$E_{(T)}$	E_{sum}
1 ↔ 2	–317	–3203	–19	–10	-3550
1 ↔ 3	–356	–3514	–19	–11	-3901
1 ↔ 4	–392	–2509	–43	–13	-2956
1 ↔ 5	–391	–2509	–43	–13	-2956
**1 ↔ 6**	**–10**	**–27**	**–5**	**–1**	**-43**
1 ↔ 7	0	–36	–1	0	-37
2 ↔ 3	–52	+393	–1	–2	337
2 ↔ 4	–27	+92	–7	–3	55
2 ↔ 5	–27	+90	–7	–3	54
**2 ↔ 6**	**–55**	**–243**	**–10**	**–4**	**-312**
2 ↔ 7	–1	+12	–1	0	10
3 ↔ 4	–32	+83	–8	–3	40
3 ↔ 5	–31	+86	–8	–3	44
3 ↔ 6	–1	–1	–1	0	-4
3 ↔ 7	–3	+12	–5	0	3
4 ↔ 5	–30	+2	–2	–2	-32
4 ↔ 6	–2	–8	–4	0	-14
4 ↔ 7	0	+3	–2	0	1
5 ↔ 6	–1	–6	–3	0	-11
5 ↔ 7	0	+3	–2	0	1
**6 ↔ 7**	**–69**	**–288**	**–29**	**–7**	**-392**

[a] Electronic preparation energies resulting from intra-fragment changes in electron density and deformation energies due to geometrical differences of fragments in interacting structure compared to their separated equilibrium geometries that are required to derive the dissociation energies corresponding to the analyzed interactions have not been included in the analysis.

Table S9. Energy components (exchange interaction, E_{exch}, electrostatic and polarization energy, E_{elstat}, dispersion interaction, E_{DISP} and contribution from triples correction, $E_{(T)}$) of the inter-fragment interaction energies (kJ mol^{-1}) in $(\mathbf{1})_2{\cdot}I_2$ structure calculated at DLPNO-CCSD(T)/def2-TZVPP level.[a]

Interaction	E_{exch}	E_{elstat}	E_{DISP}	$E_{(T)}$	E_{sum}
1 ↔ 2	–278	–3002	–17	–10	–3307
1 ↔ 3	–336	–3482	–19	–12	–3849
1 ↔ 4	–331	–2250	–36	–12	–2629
1 ↔ 5	–331	–2246	–36	–12	–2625
**1 ↔ 6**	**–11**	**–28**	**–4**	**–1**	**–44**
1 ↔ 7	0	–38	0	0	–38
2 ↔ 3	–42	+406	–1	–2	+361
2 ↔ 4	–31	+94	–6	–3	+54
2 ↔ 5	–31	+95	–6	–3	+55
**2 ↔ 6**	**–64**	**–254**	**–11**	**–4**	**–333**
2 ↔ 7	–2	+11	0	0	+9
3 ↔ 4	–37	+100	–7	–3	+53
3 ↔ 5	–37	+100	–7	–3	+53
3 ↔ 6	–2	0	–1	0	–3
3 ↔ 7	–4	+11	–3	–1	+3
4 ↔ 5	–25	+25	–1	–1	–2
4 ↔ 6	–2	–7	–3	–1	–13
4 ↔ 7	0	+3	0	0	+3
5 ↔ 6	–2	–7	–3	–1	–13
5 ↔ 7	0	+3	0	0	+3
**6 ↔ 7**	**–82**	**–295**	**–26**	**–8**	**–411**

[a] Electronic preparation energies resulting from intra-fragment changes in electron density and deformation energies due to geometrical differences of fragments in interacting structure compared to their separated equilibrium geometries that are required to derive the dissociation energies corresponding to the analyzed interactions have not been included in the analysis.

NCI-plot analysis. Experimental Procedures

All calculations were done in Critic2 program[15,23] as promolecular with nstep = 300. 3D visualizations were performed in VMD program[24] with isovalue = 0.3; colorscale data range [–3;3].

NCI-plot analysis. Results

The isosurfaces on the 3D plots are colored such that deep blue represent very strong stabilizing interactions, green represent strong and medium to weak stabilizing interactions, respectively, red represent strong destabilizing interactions. The spikes in the [–0.2, –0.05] a.u. region in all 2D plots represent polarized covalent bonds, the spikes in the [–0.04, –0.005] a.u. region in all 2D plots represent strong and medium to weak stabilizing interactions, the spikes in the [–0.005, 0] a.u. region in all 2D plots

represent weak dispersive stabilizing interactions, the spikes in the [0, 0.2] a.u. region in all 2D plots represent weak-to-strong destabilizing interactions.

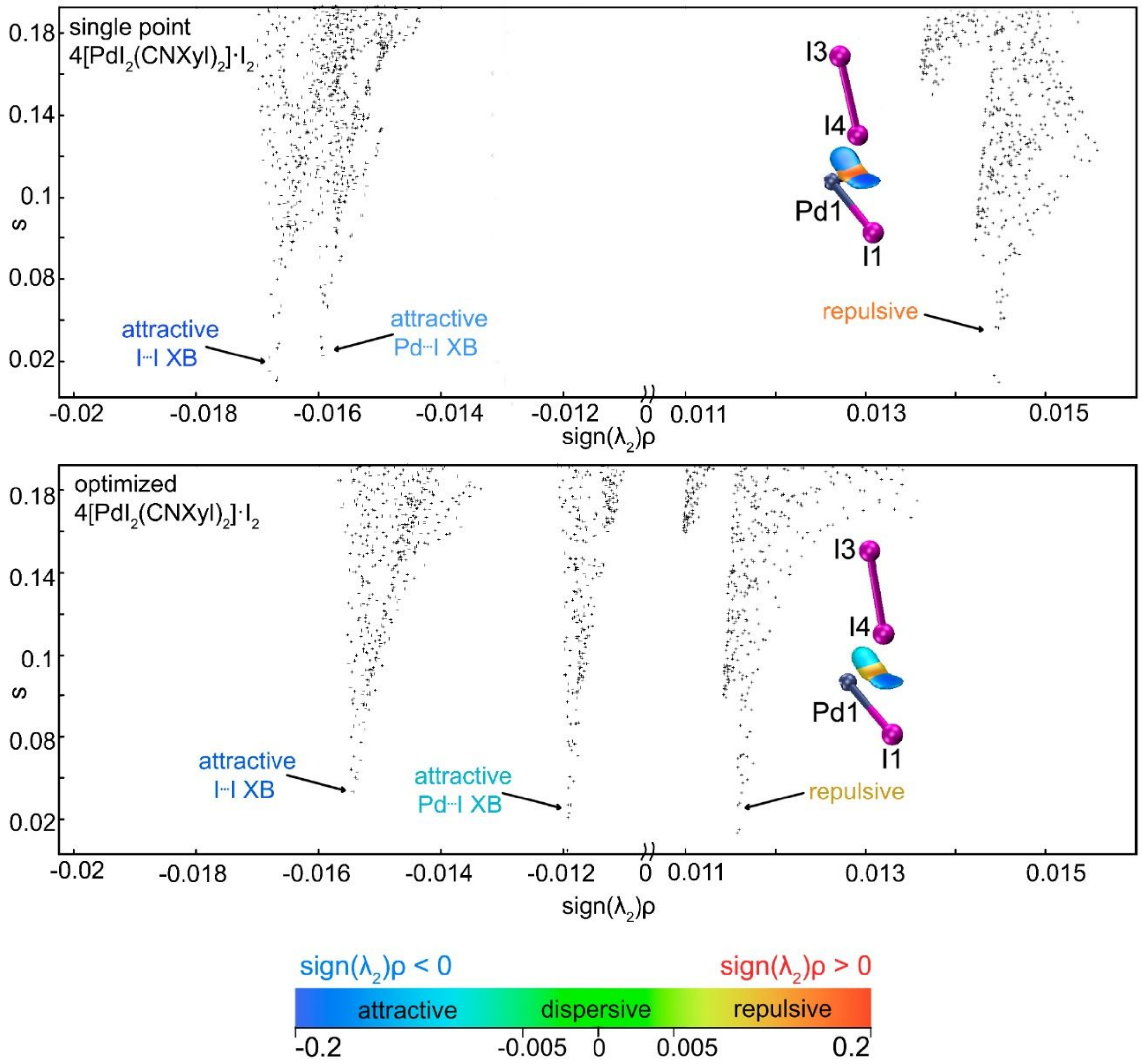

Figure S8. Combined 2D plot of the sign(λ_2)ρ × s in the [-0.02;-0.008]U[0.009;0.016] sign(λ_2)ρ a.u. range for SP (top) and OPT (bottom) $(\mathbf{1})_4\cdot I_2$ cluster with the 3D visualizations of noncovalent interactions. The 2D plot contains spikes characterizing attractive and repulsive noncovalent interactions. Only isosurfaces representing bifurcated contact are presented for clarity.

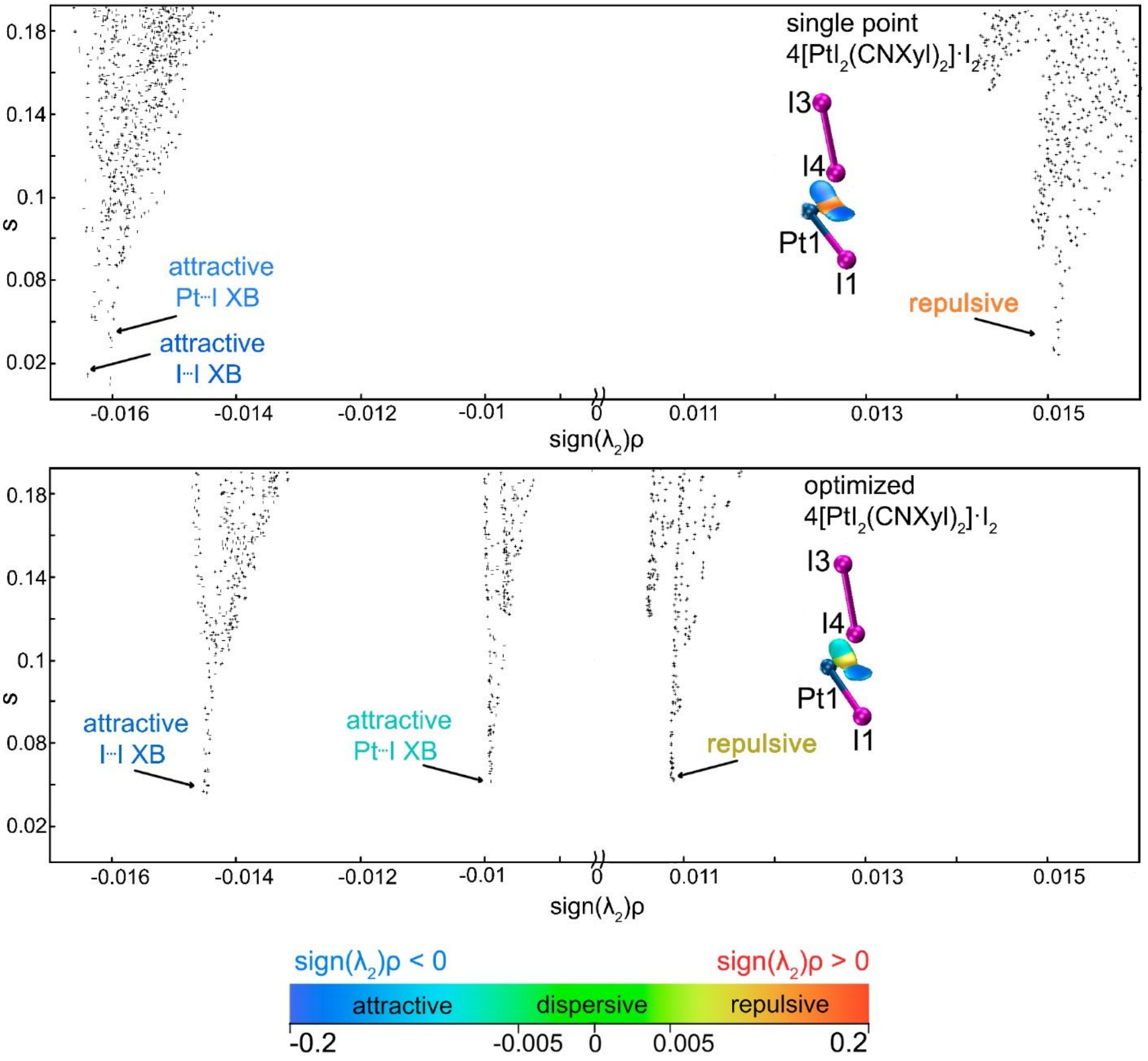

Figure S9. Combined 2D plot of the sign(λ_2)ρ × s in the [-0.017;-0.008]U[0.009;0.016] sign(λ_2)ρ a.u. range for SP (top) and OPT (bottom) $(\mathbf{2})_4{\cdot}I_2$ cluster with the 3D visualizations of noncovalent interactions. The 2D plot contains spikes characterizing attractive and repulsive noncovalent interactions. Only isosurfaces representing bifurcated contact are presented for clarity.

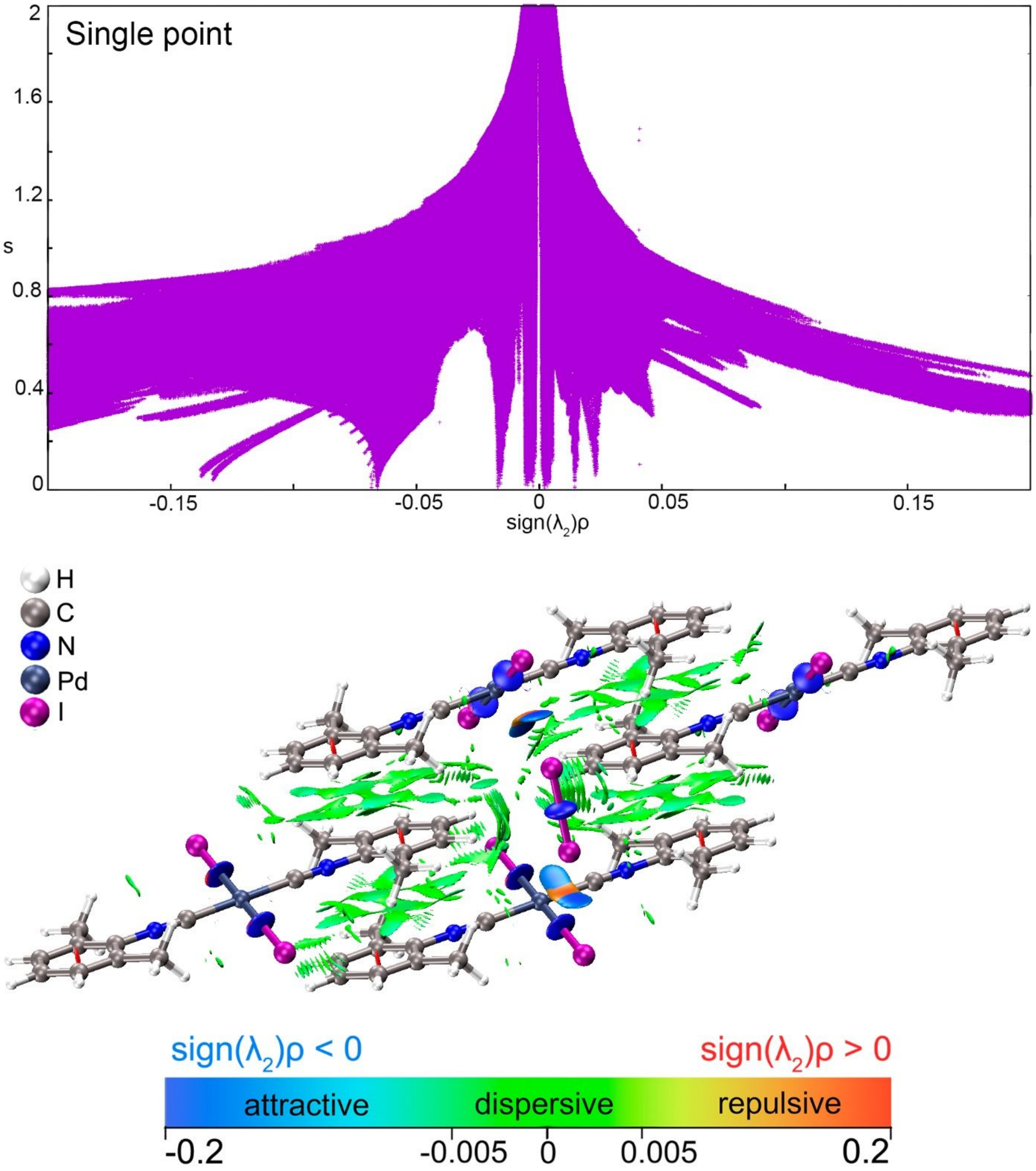

Figure S10. Isosurfaces of all NCI's calculated for single point $(\mathbf{1})_4{\cdot}I_2$ cluster. The isosurfaces are colored in a blue-green-red color scale over the range of $-0.02 < \mathrm{sign}(\lambda_2)\rho < +0.02$ a.u., s = 0.3. Dark blue and blue isosurfaces represent strong and medium stabilizing interactions, respectively, green represents dispersive interactions, and yellow/orange/red isosurfaces represent weak/medium/strong repulsive interactions, respectively.

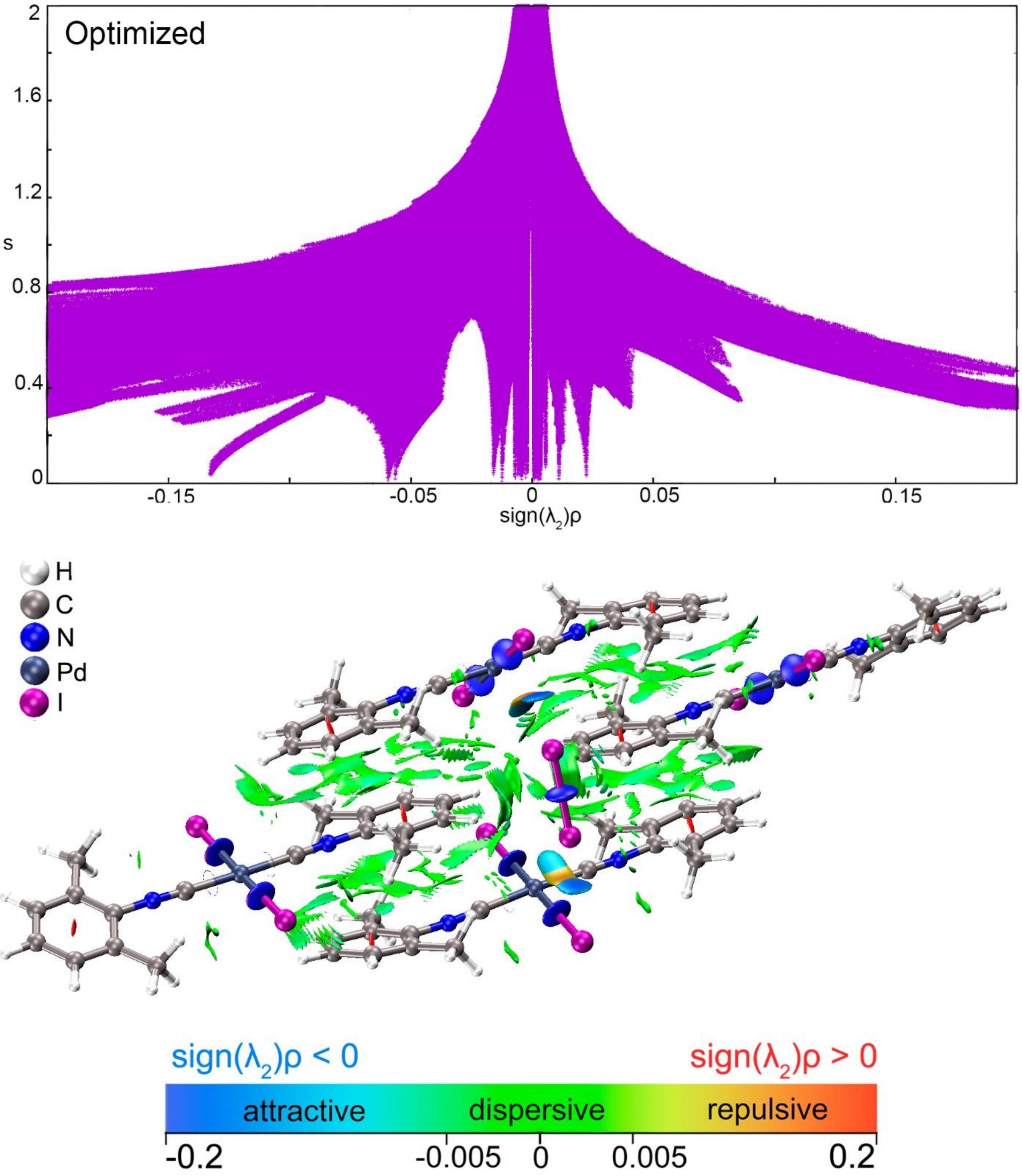

Figure S11. Isosurfaces of all NCI's calculated for optimized $(\mathbf{1})_4{\cdot}I_2$ cluster. The isosurfaces are colored in a blue-green-red color scale over the range of −0.02 < sign(λ_2)ρ < +0.02 a.u., s = 0.3. Dark blue and blue isosurfaces represent strong and medium stabilizing interactions, respectively, green represents dispersive interactions, and yellow/orange/red isosurfaces represent weak/medium/strong repulsive interactions, respectively.

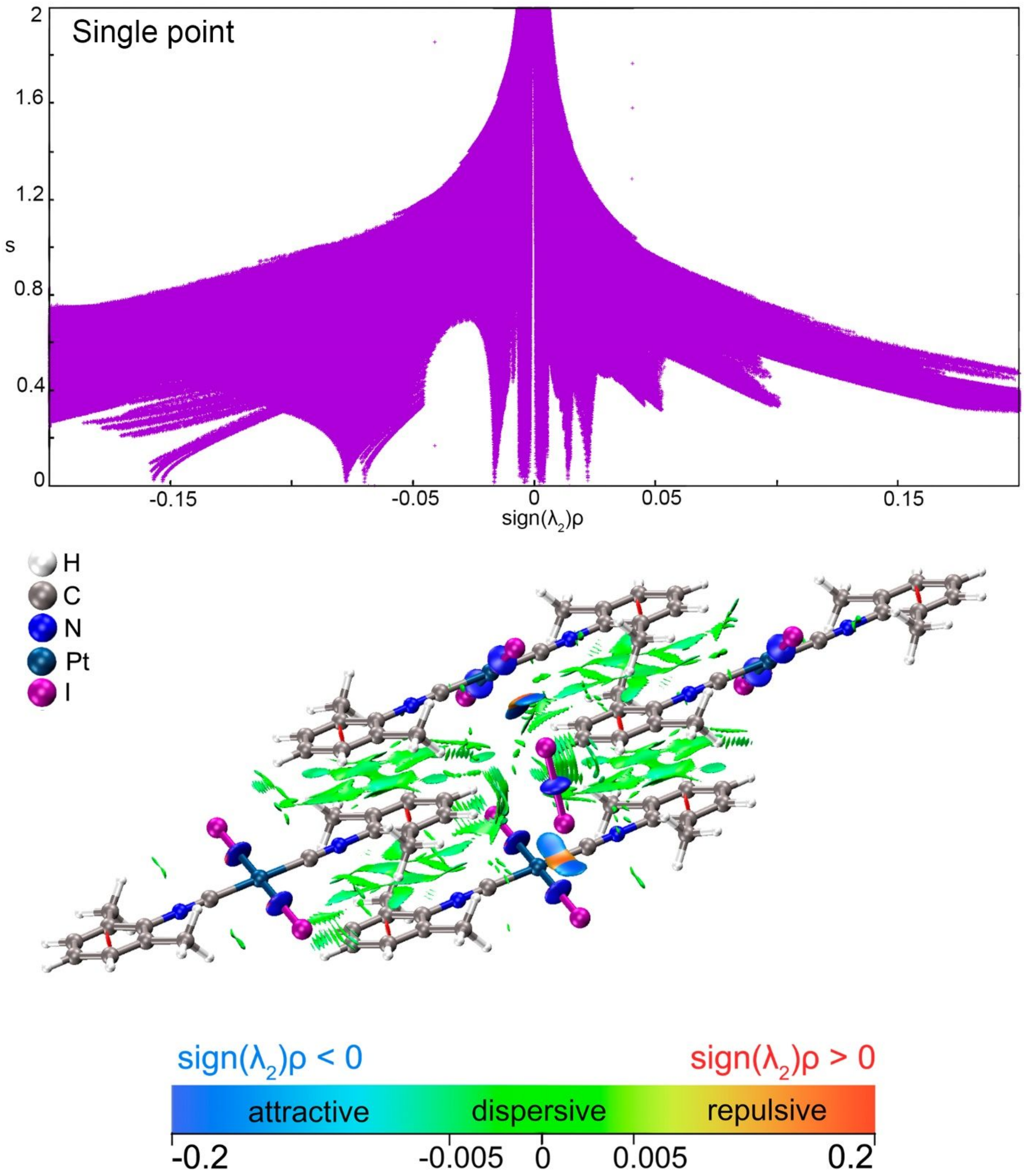

Figure S12. Isosurfaces of all NCI's calculated for single point $(\mathbf{2})_4{\cdot}I_2$ cluster. The isosurfaces are colored in a blue-green-red color scale over the range of −0.02 < sign(λ_2)ρ < +0.02 a.u., s = 0.3. Dark blue and blue isosurfaces represent strong and medium stabilizing interactions, respectively, green represents dispersive interactions, and yellow/orange/red isosurfaces represent weak/medium/strong repulsive interactions, respectively.

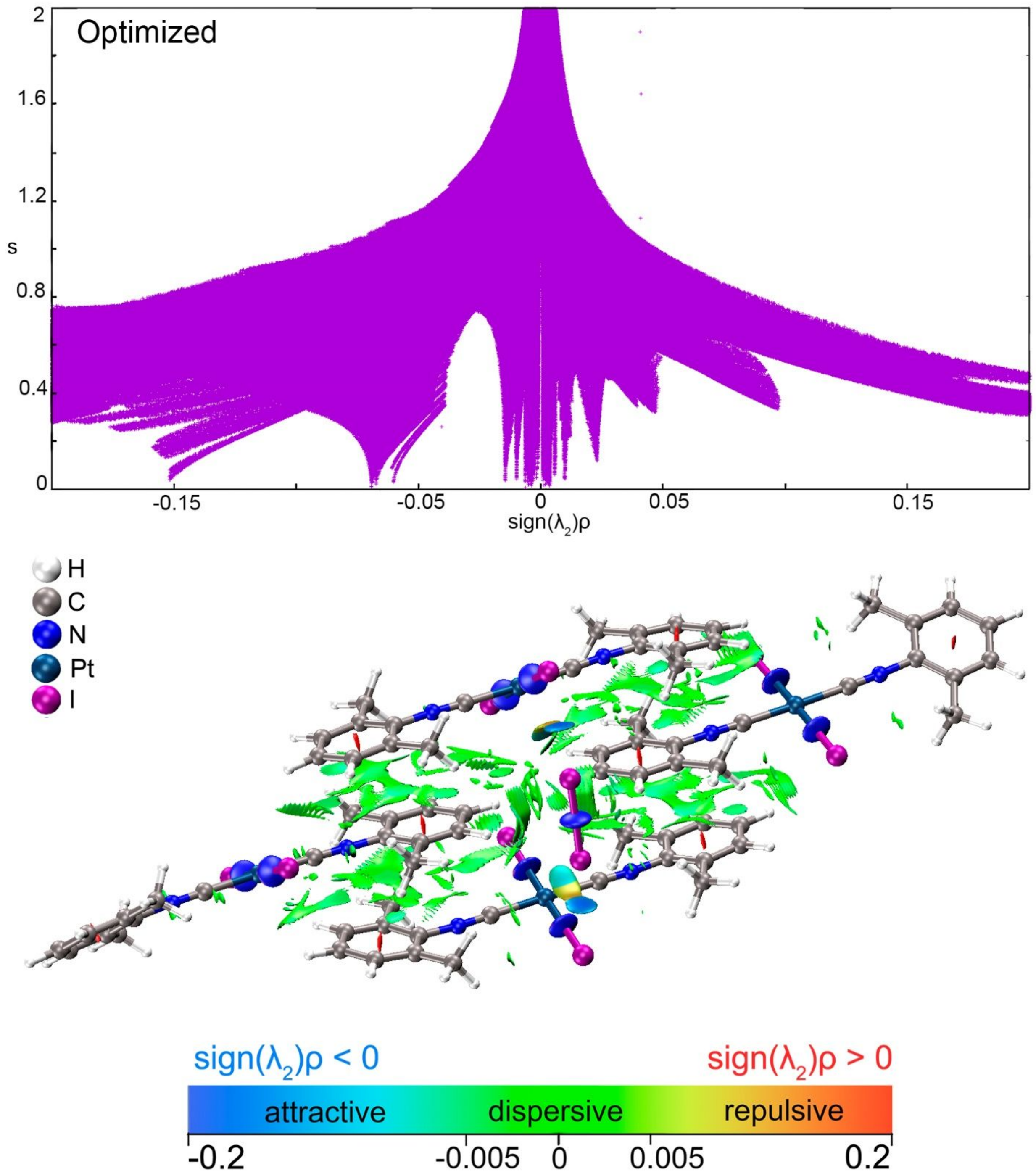

Figure S13. Isosurfaces of all NCI's calculated for optimized $(\mathbf{2})_4{\cdot}I_2$ cluster. The isosurfaces are colored in a blue-green-red color scale over the range of −0.02 < sign(λ_2)ρ < +0.02 a.u., s = 0.3. Dark blue and blue isosurfaces represent strong and medium stabilizing interactions, respectively, green represents dispersive interactions, and yellow/orange/red isosurfaces represent weak/medium/strong repulsive interactions, respectively.

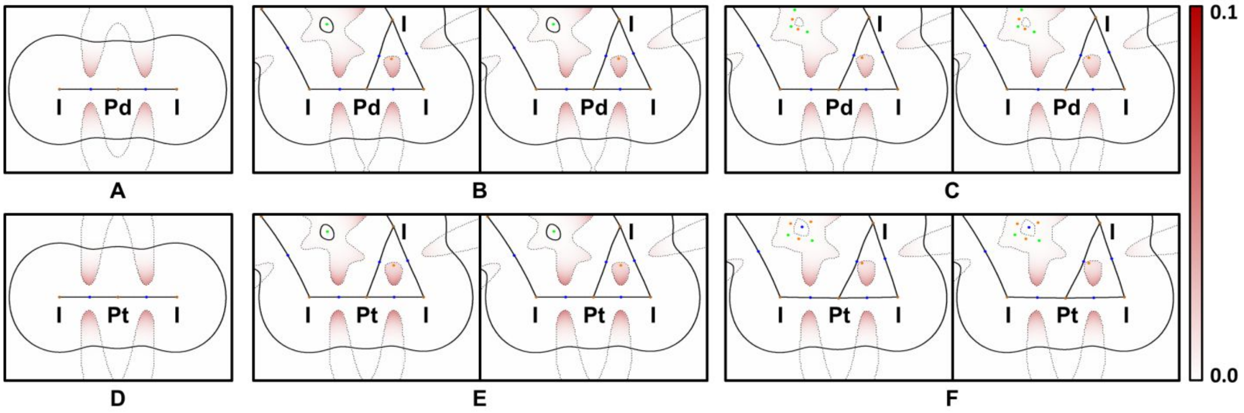

Figure S14. Sign(λ_2)ρ projections through **I**−M−**I** lines and normal to the NC−M planes with plotted contour line (Sign(λ_2)ρ = 0 a.u.), bond paths (black lines), ρ = 0.001 a.u. black line, bond critical points (blue dots), nuclear critical points (brown dots), ring critical points (orange dots), and cage critical points (green dots) in the OPT **1** (**A**), SP ($\mathbf{1}$)$_4$·I_2 (**B**), OPT ($\mathbf{1}$)$_4$·I_2 (**C**), OPT **2** (**D**), SP ($\mathbf{2}$)$_4$·I_2 (**E**), and OPT ($\mathbf{2}$)$_4$·I_2 (**F**) model clusters. Sign(λ_2)ρ units are a. u.

Analysis of electron localization function (ELF) and ED/ESP minima

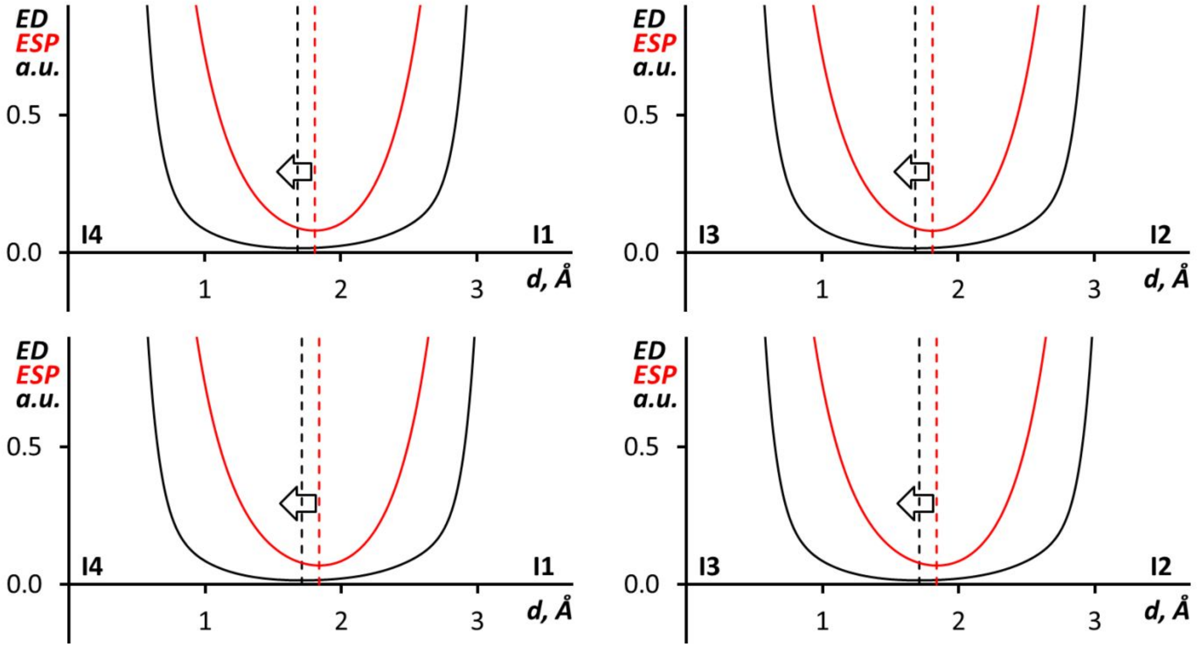

Figure S15. 1D profiles of the ED (black) and ESP (red) functions along the I···I bond paths in ($\mathbf{1}$)$_4$·I_2 for SP (upper graphs) and OPT (lower graphs) structures.

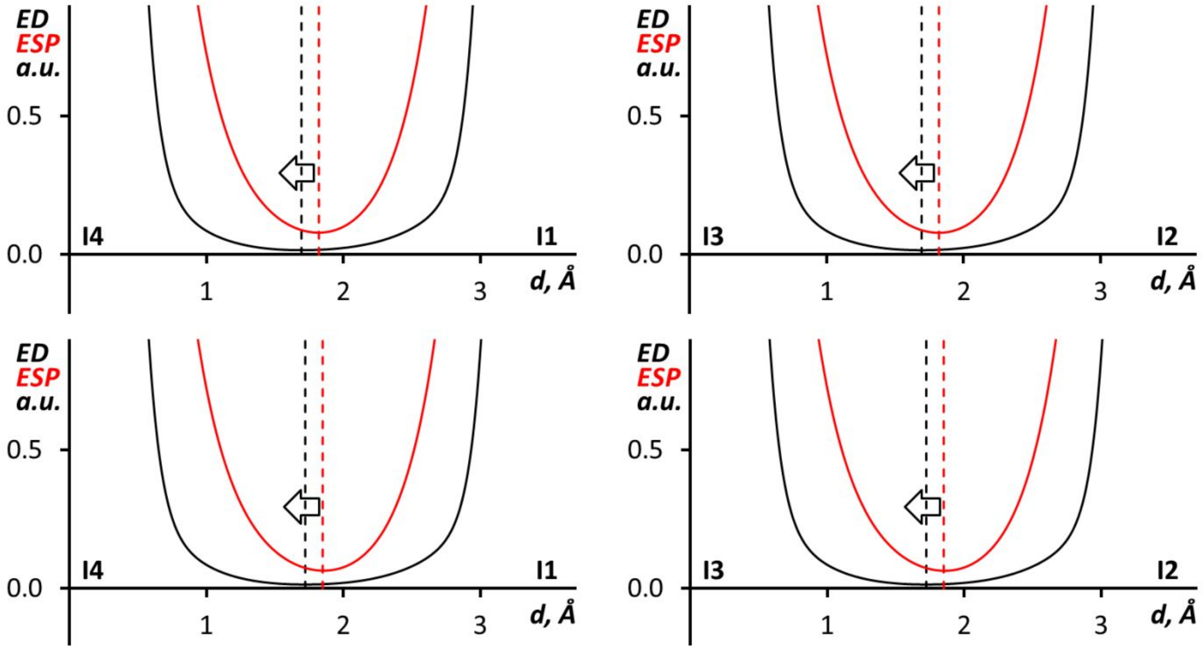

Figure S16. 1D profiles of the ED (black) and ESP (red) functions along the I···I bond paths in (**2**)$_4$·I$_2$ for SP (upper graphs) and OPT (lower graphs) structures.

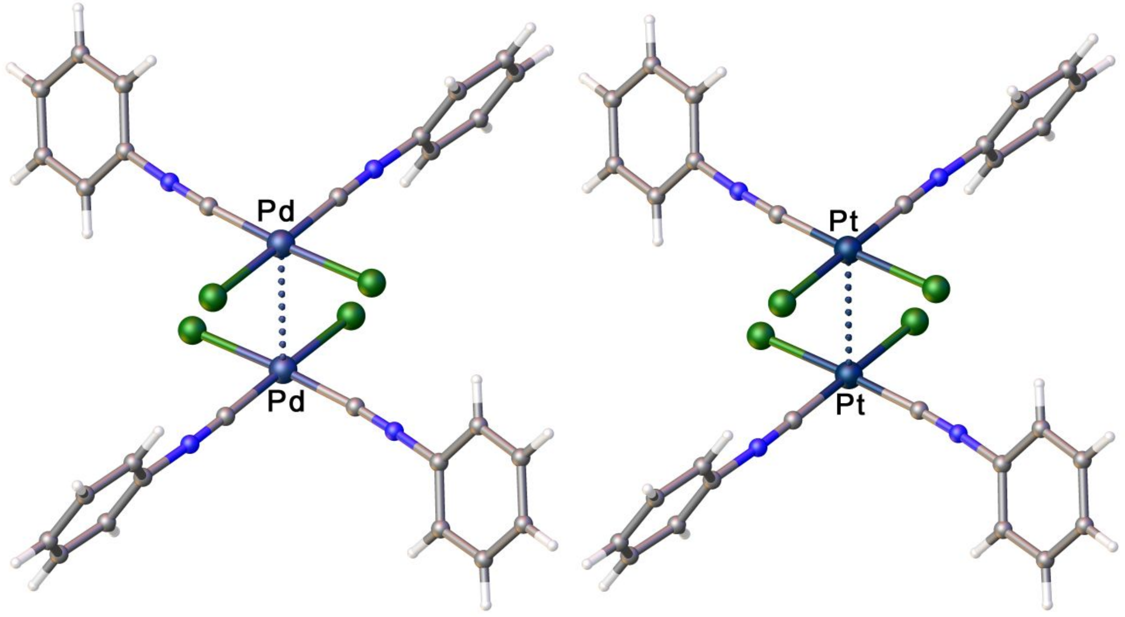

Figure S17. View of (*cis*-[PdCl$_2$(CNPh)$_2$])$_2$ (left) and (*cis*-[PtCl$_2$(CNPh)$_2$])$_2$ (right) model clusters.

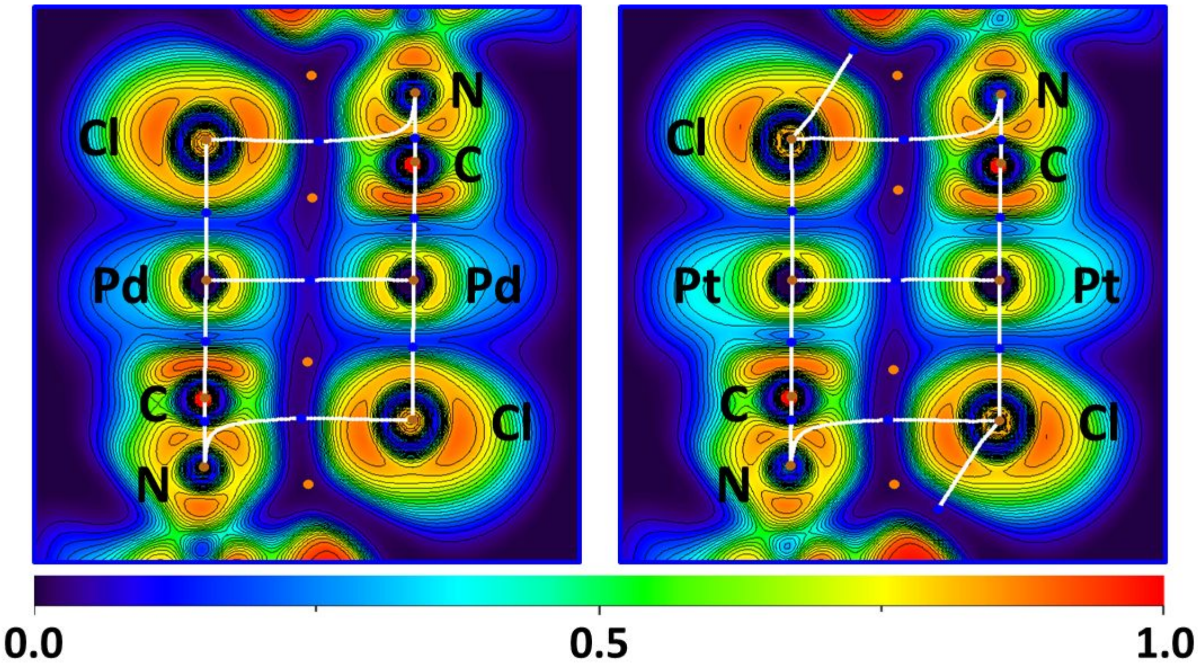

Figure S18. ELF projections with plotted contour lines (black, step is 0.05), bond paths (white lines), bond critical points (blue dots), nuclear critical points (brown dots), and ring critical points (orange dots) for the M···M interactions in (*cis*-[PdCl$_2$(CNPh)$_2$])$_2$ (left) and (*cis*-[PtCl$_2$(CNPh)$_2$])$_2$ (right) model clusters.

References

(1) Rigaku Oxford Diffraction. CrysAlisPro Software System. Rigaku Corporation: Oxford, UK. 2017.

(2) Sheldrick, G. M. A Short History of SHELX. *Acta Crystallogr. Sect. A* **2008**, *64* (1), 112–122. https://doi.org/10.1107/S0108767307043930.

(3) Sheldrick, G. M. SHELXL13. Program Package for Crystal Structure Determination from Single Crystal Diffraction Data. University of Göttingen, Germany 2013.

(4) Thorn, A.; Dittrich, B.; Sheldrick, G. M. Enhanced Rigid-Bond Restraints. *Acta Crystallogr. Sect. A* **2012**, *68* (4), 448–451. https://doi.org/10.1107/S0108767312014535.

(5) Hirshfeld, F. L. Can X-Ray Data Distinguish Bonding Effects from Vibrational Smearing? *Acta Crystallogr. Sect. A* **1976**, *32* (2), 239–244. https://doi.org/10.1107/S0567739476000533.

(6) Sheldrick, G. M. Crystal Structure Refinement with SHELXL. *Acta Crystallogr. Sect. C Struct. Chem.* **2015**, *71*, 3–8. https://doi.org/10.1107/S2053229614024218.

(7) Degen, T.; Sadki, M.; Bron, E.; König, U.; Nénert, G. The High Score Suite. In *Powder Diffraction*; Cambridge University Press, 2014; Vol. 29, pp S13–S18. https://doi.org/10.1017/S0885715614000840.

(8) Pawley, G. S. Unit-Cell Refinement from Powder Diffraction Scans. *J. Appl. Crystallogr.* **1981**, *14* (6), 357–361. https://doi.org/10.1107/S0021889881009618.

(9) Frisch, M. J.; Trucks, G. W.; Schlegel, H. B.; Scuseria, G. E.; Robb, M. A.; Cheeseman, J. R.; Scalmani, G.; Barone, V.; Petersson, G. A.; Nakatsuji, H.; Li, X.; Caricato, M.; Marenich, A. V; Bloino, J.; Janesko, B. G.; Gomperts, R.; Mennucci, B.; Hratchian, H. P.; Ortiz, J. V; Izmaylov, A. F.; Sonnenberg, J. L.; Williams-Young, D.; Ding, F.; Lipparini, F.;

Egidi, F.; Goings, J.; Peng, B.; Petrone, A.; Henderson, T.; Ranasinghe, D.; Zakrzewski, V. G.; Gao, J.; Rega, N.; Zheng, G.; Liang, W.; Hada, M.; Ehara, M.; Toyota, K.; Fukuda, R.; Hasegawa, J.; Ishida, M.; Nakajima, T.; Honda, Y.; Kitao, O.; Nakai, H.; Vreven, T.; Throssell, K.; Montgomery Jr., J. A.; Peralta, J. E.; Ogliaro, F.; Bearpark, M. J.; Heyd, J. J.; Brothers, E. N.; Kudin, K. N.; Staroverov, V. N.; Keith, T. A.; Kobayashi, R.; Normand, J.; Raghavachari, K.; Rendell, A. P.; Burant, J. C.; Iyengar, S. S.; Tomasi, J.; Cossi, M.; Millam, J. M.; Klene, M.; Adamo, C.; Cammi, R.; Ochterski, J. W.; Martin, R. L.; Morokuma, K.; Farkas, O.; Foresman, J. B.; Fox, D. J. Gaussian 16 Revision C.01. Gaussian Inc. Wallingford CT 2016.

(10) Zhao, Y.; Truhlar, D. G. A New Local Density Functional for Main-Group Thermochemistry, Transition Metal Bonding, Thermochemical Kinetics, and Noncovalent Interactions. *J. Chem. Phys.* **2006**, *125* (19), 194101. https://doi.org/10.1063/1.2370993.

(11) Andrae, D.; Häußermann, U.; Dolg, M.; Stoll, H.; Preuß, H. Energy-Adjustedab Initio Pseudopotentials for the Second and Third Row Transition Elements. *Theor. Chim. Acta* **1990**, *77* (2), 123–141. https://doi.org/10.1007/BF01114537.

(12) Peterson, K. A.; Figgen, D.; Goll, E.; Stoll, H.; Dolg, M. Systematically Convergent Basis Sets with Relativistic Pseudopotentials. II. Small-Core Pseudopotentials and Correlation Consistent Basis Sets for the Post-d Group 16–18 Elements. *J. Chem. Phys.* **2003**, *119* (21), 11113–11123. https://doi.org/10.1063/1.1622924.

(13) Weigend, F.; Ahlrichs, R. Balanced Basis Sets of Split Valence, Triple Zeta Valence and Quadruple Zeta Valence Quality for H to Rn: Design and Assessment of Accuracy. *Phys. Chem. Chem. Phys.* **2005**, *7*, 3297–3305. https://doi.org/10.1039/b508541a.

(14) Contreras-García, J.; Johnson, E. R.; Keinan, S.; Chaudret, R.; Piquemal, J.-P.; Beratan, D. N.; Yang, W. NCIPLOT: A Program for Plotting Non-Covalent Interaction Regions. *J. Chem. Theory Comput.* **2011**, *7* (3), 625–632. https://doi.org/10.1021/ct100641a.

(15) Otero-de-la-Roza, A.; Johnson, E. R.; Luaña, V. Critic2: A Program for Real-Space Analysis of Quantum Chemical Interactions in Solids. *Comput. Phys. Commun.* **2014**, *185* (3), 1007–1018. https://doi.org/10.1016/J.CPC.2013.10.026.

(16) Keith, T. A. AIMALL (Version 12.06.03),. *TK Gristmill Software, Overland Park KS, USA*. 2003.

(17) Lu, T.; Chen, F. Multiwfn: A Multifunctional Wavefunction Analyzer. *J. Comput. Chem.* **2012**, *33* (5), 580–592. https://doi.org/10.1002/jcc.22885.

(18) Fabijańska, M.; Orzechowska, M.; Rybarczyk-Pirek, A. J.; Dominikowska, J.; Bieńkowska, A.; Małecki, M.; Ochocki, J. Simple Trans-Platinum Complex Bearing 3-Aminoflavone Ligand Could Be a Useful Drug: Structure-Activity Relationship of Platinum Complex in Comparison with Cisplatin. *Int. J. Mol. Sci.* **2020**, *21* (6). https://doi.org/10.3390/ijms21062116.

(19) Xu, X.; Pooi, B.; Hirao, H.; Hong, S. H. CH–π and CF–π Interactions Lead to Structural Changes of N-Heterocyclic Carbene Palladium Complexes. *Angew. Chem., Int. Ed.* **2014**, *53* (5), 1283–1287. https://doi.org/https://doi.org/10.1002/anie.201309371.

(20) Hosseinnejad, T.; Kazemi, T. Quantum Chemical Investigation on Complexation of Palladium with Iminopyridyl Ligands: Structural, Thermochemical, and Electronic Aspects. *Mol. Cryst. Liq. Cryst.* **2016**, *637* (1), 53–64. https://doi.org/10.1080/15421406.2016.1206807.

(21) Cremer, D.; Kraka, E. A Description of the Chemical Bond in Terms of Local Properties of Electron Density and Energy. *Croat. Chem. Acta* **1984**, *57* (6).

(22) A. Cabeza, J.; F. Van der Maelen, J.; García-Granda, S. Topological Analysis of the Electron Density in the N-Heterocyclic Carbene Triruthenium Cluster [Ru3(μ-H)2(M3-

MeImCH)(CO)9] (Me2Im = 1,3-Dimethylimidazol-2-Ylidene). *Organometallics* **2009**, *28* (13), 3666–3672. https://doi.org/10.1021/om9000617.

(23) Otero-de-la-Roza, A.; Blanco, M. A.; Pendás, A. M.; Luaña, V. Critic: A New Program for the Topological Analysis of Solid-State Electron Densities. *Comput. Phys. Commun.* **2009**, *180* (1), 157–166. https://doi.org/10.1016/J.CPC.2008.07.018.

(24) Humphrey, W.; Dalke, A.; Schulten, K. VMD: Visual Molecular Dynamics. *J. Mol. Graph.* **1996**, *14* (1), 33–38. https://doi.org/10.1016/0263-7855(96)00018-5.
